# Hierarchical modeling of tumor subtypes in cell lines using large-scale genomic datasets

**DOI:** 10.1016/j.isci.2026.117315

**Published:** 2026-08-25

**Authors:** Juho Mikkonen, Teemu J. Rintala, Vittorio Fortino

**Affiliations:** 1 Institute of Biomedicine, University of Eastern Finland, 70210 Kuopio, Finland

**Keywords:** machine learning, cancer cell lines, transcriptomics, domain alignment, hierarchical classification, subtype representativeness

## Abstract

Cancer cell lines (CLs) are widely used to study tumor biology and drug response, yet their translational relevance is often limited by inaccurate subtype annotations. Existing CL-tumor matching approaches are frequently constrained by flat classification schemes, weak subtype definitions, and the exclusion of normal tissue references, leading to potential confounding of tumor-specific and tissue-of-origin signals. To address these limitations, a hierarchical classification (HC) framework is presented in which CLs are aligned with patient tumors across biological resolutions, from organ to molecular subtype. Gene expression profiles from 802 CLs, 5,612 tumors from The Cancer Genome Atlas (TCGA) , and 8,939 non-cancerous tissues were integrated to separate oncogenic signals from tissue-specific signals. Node-specific features were selected using maximum relevance minimum redundancy, and balanced accuracies of 89% in cross-validation and 75%, and 80% on external datasets were achieved. Through the framework, 43 CLs were reassigned, and clinically relevant underrepresented subtypes were identified.

## Highlights

• Cancer cell lines often lack robust and clinically relevant tumor subtype labels
• Hierarchical models assigned tissue, cancer type, and subtype identities
• Several cell lines were not representative of any related tumor subtypes
• Many tumor subtypes remain poorly represented by existing cell line panels

## Graphical abstract

**Figure:**
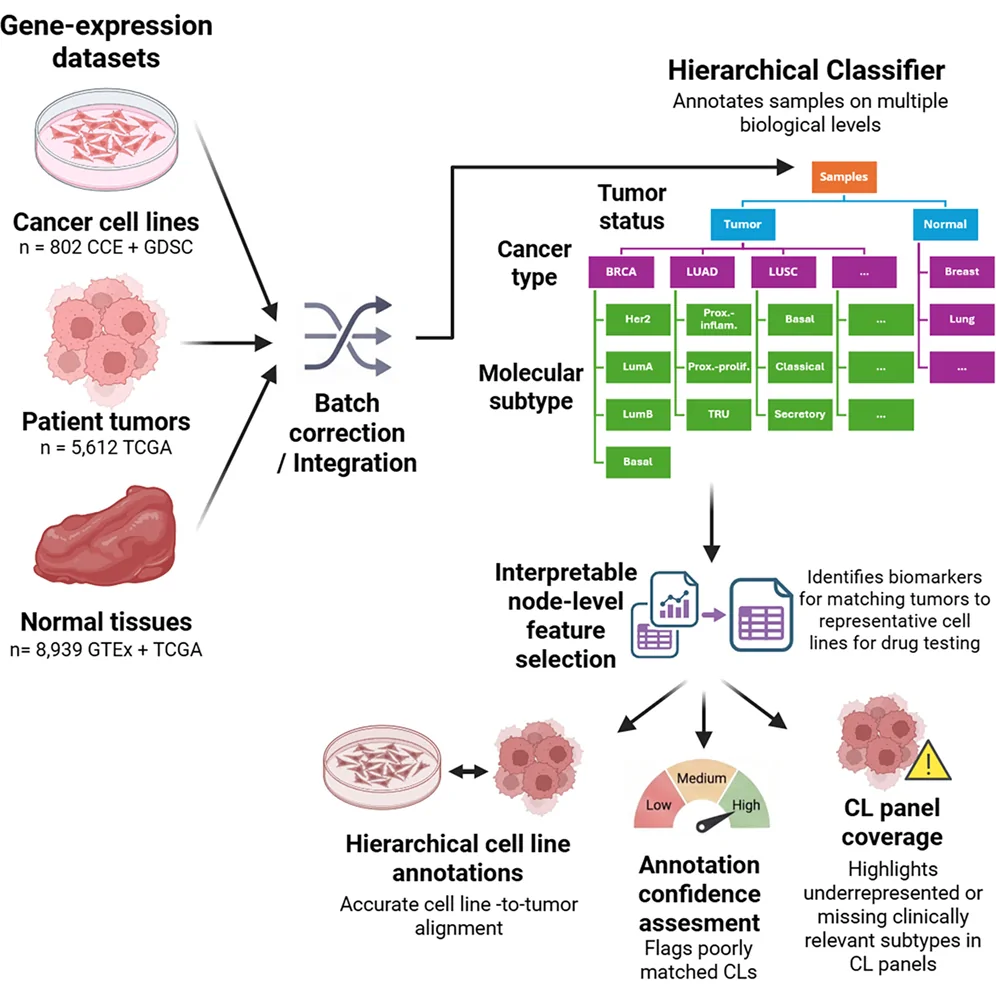

## Teaser

Bioinformatics; Cancer; Machine learning

## Introduction

Cell lines (CLs) are efficient tools for studying biological mechanisms, drug responses, and gene function while avoiding the complexities of primary tissues and whole organisms. They are cost-effective, consistent, readily scalable, and amenable to high-throughput sequencing. As a result, large resources such as the Cancer Cell Line Encyclopedia (CCLE) and the Genomics of Drug Sensitivity in Cancer (GDSC) link multi-omics readouts with responses to anticancer agents across diverse cancer types and subtypes, offering opportunities for precision medicine by informing therapy choices from molecular profiles.^1^ Despite this promise, the clinical translatability of CL resources remains uncertain. CLs are typically used under the assumption that they preserve the genomic and epigenomic features of the tumors they model. In practice, they may diverge during derivation and passaging through genetic drift, clonal selection, or overgrowth of unrepresentative populations.^2^ Additionally, original sample mislabeling due to clinician errors, contamination, or sampling from metastatic lesions has been reported.^3^ As a result, the nominal annotations of CLs cannot be assumed to reliably reflect their true tumor identity, creating the need to develop algorithms that address this technical gap by improving the alignment between *in vitro* cancer CL models and available cancer patient data. Such misalignment may have important consequences, particularly for the use of high-throughput drug screening data generated *in vitro*. Therapeutic vulnerabilities identified in these drug screens can only be reliably extrapolated to cancer patients if the models accurately represent the tumor subtype and molecular phenotype of their tumors of origin. Alignment between cancer CLs and patient tumors is particularly relevant for downstream pharmacogenomic applications, including machine learning-based drug sensitivity prediction. Recent studies integrating transcriptomic and chemical features have shown that model performance strongly depends on the biological fidelity of preclinical models, further supporting the importance of robust CL annotation frameworks.^4^

Previous studies have leveraged bulk RNA sequencing (RNA-seq) data from The Cancer Genome Atlas (TCGA) patients and major CL resources such as CCLE and GDSC to improve the mapping of CLs to their tumors and subtypes of origin. Machine-learning approaches have been used to model high-dimensional gene expression patterns, often by combining batch-correction methods such as mutual nearest neighbors or ComBat with supervised classifiers, or alternatively by utilizing binary event matrices (e.g., somatic mutations) to avoid explicit batch correction.^5^^,^^6^^,^^7^ Building on this foundation, extensive work has aimed to clarify the cellular origins of CLs and evaluate how well they preserve tumor characteristics *in vitro*. Cancer type-specific studies have shown that many CLs can be assigned to subtypes, while others show characteristics inconsistent with their annotations.^8^^,^^9^^,^^10^^,^^11^ Studies using pan-cancer models trained on TCGA and applied to CCLE have proposed curated CL panels and identified CLs more closely related to other cancer types than originally suggested.^5^^,^^6^^,^^12^^,^^13^^,^^14^ Additionally, multi-omics similarity-based approaches, such as that of Bose and Bozdag,^15^ and integrative methods like Salvadores et al.,^5^ have provided further insight into misannotated CLs and refined CL annotations.

Despite previous efforts to improve the alignment between cancer CLs and patient tumors, important technical gaps remain. First, tumor classification is most often formulated in a one-versus-all manner, treating cancer types and subtypes as independent and equally distinct categories and failing to account for obvious biological relationships among them. Second, most approaches operate on genome-wide expression profiles without explicit feature selection, allowing technical or cell-culture-related signals to dominate alignment and classification. Third, normal tissues are typically excluded from these analyses, limiting biological resolution and making it difficult to determine whether molecular features driving classification are truly oncogenic or instead reflect normal tissue identity. This distinction is critical for identifying disease relevant markers and improving model interpretability.

In this study, these technical gaps were addressed through multiple improvements. First, clinically meaningful cancer subtypes were manually curated to increase biological and clinical relevance. Secondly, to enable robust comparisons between cancer CLs and patient tissue samples, gene expression data from TCGA, The Genotype-Tissue Expression project (GTEx), CCLE, and GDSC were integrated and batch correction was applied using ComBat. Importantly, this approach incorporated non-cancerous tissue profiles from GTEx and TCGA adjacent samples, providing a biologically grounded reference for distinguishing cancer-specific molecular alterations from tissue-of-origin effects. Third, CL profiles from GDSC were included in addition to those from CCLE, expanding the diversity and number of CLs analyzed. Fourth, a hierarchical classification (HC) framework was developed to model the nested organization of cancer (organ, cancer type, and subtype), reducing biologically implausible cross-organ confusions and improving interpretability. Finally, node-specific feature selection was applied along the hierarchy to identify informative genes at each decision point, providing insight into molecular determinants of tissue specificity and subtype identity.

Using the framework, gene expression profiles from GTEx, TCGA, CCLE, and GDSC were aligned through batch correction, leveraging non-cancerous tissue samples from GTEx and TCGA tumor adjacent samples to establish a robust tissue baseline. The HC models were then trained on 8,939 non-cancerous and 5,612 tumor samples spanning 12 organs and 18 TCGA cancer types. The trained models were subsequently applied to predict organ, cancer type, and molecular subtype for 802 cancer CL gene expression profiles from CCLE and GDSC. To further evaluate model robustness, generalizability was assessed using an external ovarian cancer (OV) dataset and a breast cancer pseudobulk dataset composed exclusively of tumor cells, providing a CL-like validation context. Finally, applicability domain (AD) and prediction confidence metrics were used to assess the reliability of individual predictions, supporting transparent downstream use of the annotations.

## Results

### Incorporating normal tissues into batch correction improves alignment of cancer CLs with tumor expression landscapes

The first objective of this study was to improve the alignment between gene expression profiles of cancer CLs and those of cancer patients by explicitly incorporating normal tissue references.

**Figure 1:**
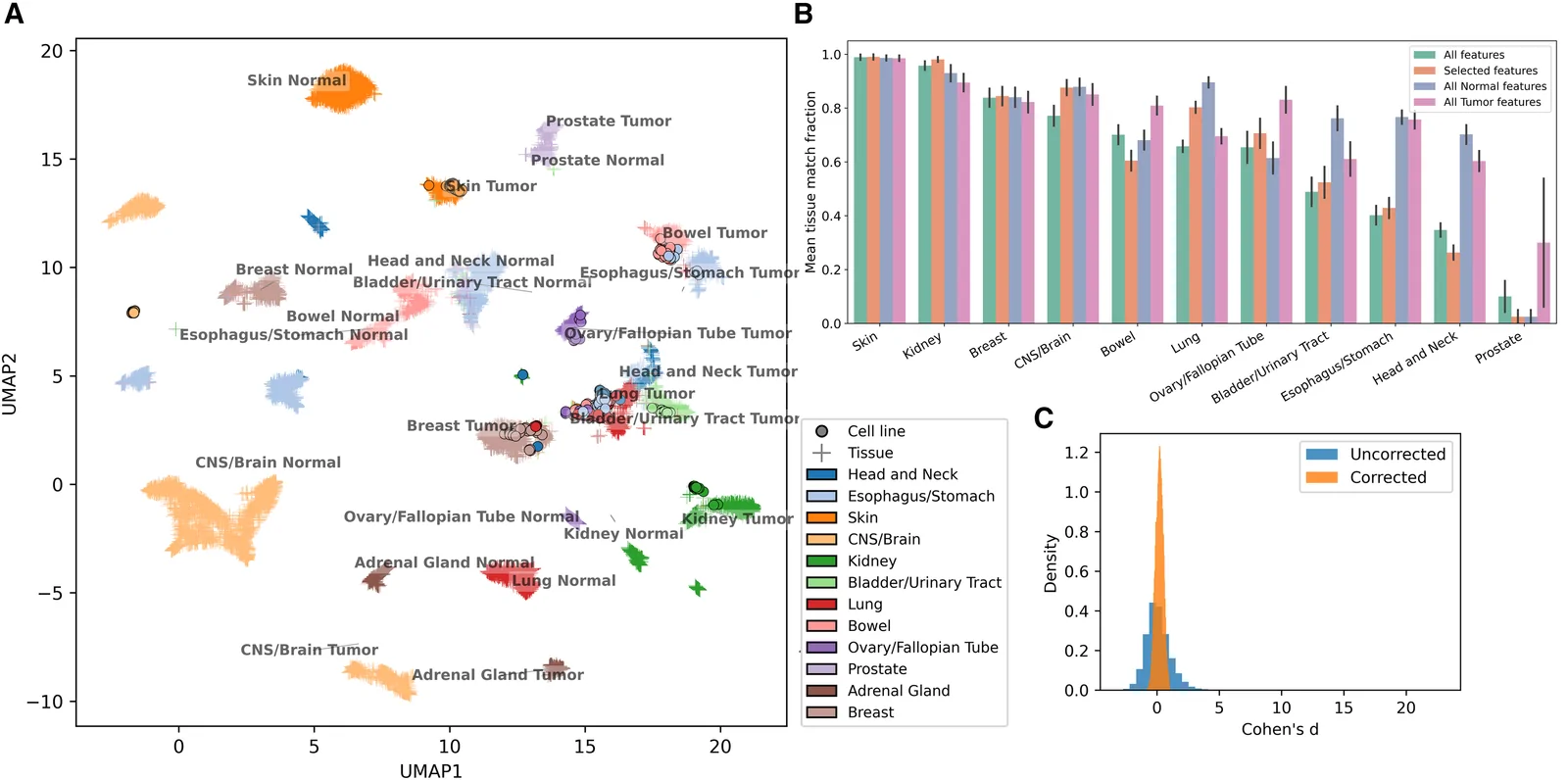
Alignment of CL and patient sample gene expression profiles (A) Uniform manifold approximation and projection (UMAP) for batch corrected (ComBat) log2-transformed transcripts per million (TPM) values for patient tissue samples (+) and CL samples (o). (B) Mean lineage match of CLs and their 10 nearest neighbor patient samples using all 13,007 features, all features selected by the best model, all normal-node features and all tumor-node features in PCA space (20 PCs) (error bars = standard error). (C) The effect sizes (Cohen’s d) of all genes between patients and CLs for batch corrected and uncorrected data (normalized).

As demonstrated in Figure 1, the batch correction framework yielded respectable alignment between CLs and their corresponding tissue origins. Prior to batch correction, only skin and esophagus/stomach CLs aligned with patient samples (Figure S2). Post-correction, CLs clustered more closely with their supposed tumor samples they are intended to represent, particularly for breast, colorectal, skin, kidney, ovary/fallopian tube, and bladder cancers. Notably, esophagus and stomach CLs overlapped with bowel tumor samples, supporting the choice of grouping these cancers under shared gastrointestinal (GI) subtypes. This overlap reflects the underlying molecular similarity across GI cancers and their CLs and justifies the use of a unified subtype classification for these tissues in downstream analyses. A subset of CLs also aligned with lung tumors, especially those derived from squamous epithelium (e.g., head and neck squamous cell carcinoma [HNSC] and esophageal squamous cell carcinoma [ESCC]), which likely reflects their similarity to lung squamous cell carcinoma (LUSC) (Figure 1A). Brain cancer CLs formed a distinct group from both patient brain tumors and other CLs, reflecting unique expression characteristics while still remaining most similar to brain tumors.

CL-tumor alignment was quantified using the tissue match fraction based on the 10 nearest patient neighbors in principal component analysis (PCA) space (Figure 1B). Restricting the analysis to features selected by the best HC model improved tissue-level matching relative to the full gene set. The largest improvements were observed for lung (0.658–0.774), CNS/brain (0.772–0.851), and ovary/fallopian tube cancers (0.654–0.706). Poor matching was observed for immortalized non-cancerous prostate CL profiles, with match fractions of 0.100 using all genes and 0.025 using the selected feature set. Across all cancer types, the mean tissue match fraction increased from 0.678 using all genes to 0.688 using HC-selected features, further increasing to 0.772 and 0.801 when restricting the analysis to features selected for TCGA type discrimination and non-cancerous lineage separation, respectively.

Effect size analysis using Cohen’s d demonstrated that ComBat correction substantially reduced gene expression differences between CL and patient samples (Figure 1C). Following batch correction, separation between CL and tissue samples was no longer possible using a logistic classifier (balanced accuracy = 0.50), whereas patient sex remained predictable in CLs when models were trained on patient tissue data (balanced accuracy = 0.77), indicating preservation of biologically meaningful signals.

Ablation benchmarking showed that including normal tissue samples during training improved cross-validation (CV) balanced accuracy, particularly when combined with ComBat batch correction and covariates. Additionally, batch correction did not artificially inflate performance when predictions were restricted to TCGA tumor samples (Figure S3A). In the external breast cancer pseudobulk dataset, batch correction was critical for maintaining predictive performance and substantially improved balanced accuracy (Figure S3B). In contrast, including normal samples provided only modest additional benefits. These results indicate that batch correction is necessary for robust cross-dataset generalization, while including normal samples primarily contributes with smaller performance gains.

Overall, batch effects between datasets were effectively minimized while retaining biological variation. Complete homogenization was not achieved, reflecting inherent biological differences among datasets, with GTEx and pseudobulk samples remaining the most distinct, CCLE and GDSC samples exhibiting the highest similarity, and TCGA samples showing the closest correspondence to CL profiles.

### HC enhances tumor subtype assignment accuracy

CLs were mapped to their tumor and subtype of origin using models trained on TCGA samples and their corresponding molecular subtypes. Therefore, four hierarchical designs for assigning the correct cancer subtype labels to TCGA patient samples were implemented and evaluated in combination with six inner classifiers (LogReg with L1, L2, Elastic Net, or no penalty; support vector machine [SVM] with linear or radial basis function [RBF] kernels) using nested CV. Inner folds were used for hyperparameter tuning and feature selection, while outer folds provided an unbiased estimate of generalization performance. In addition, external validation was performed using pseudobulk transcriptomic data from breast cancer patients derived from single-cell datasets to evaluate classification accuracy in an independent setting.

**Figure 2:**
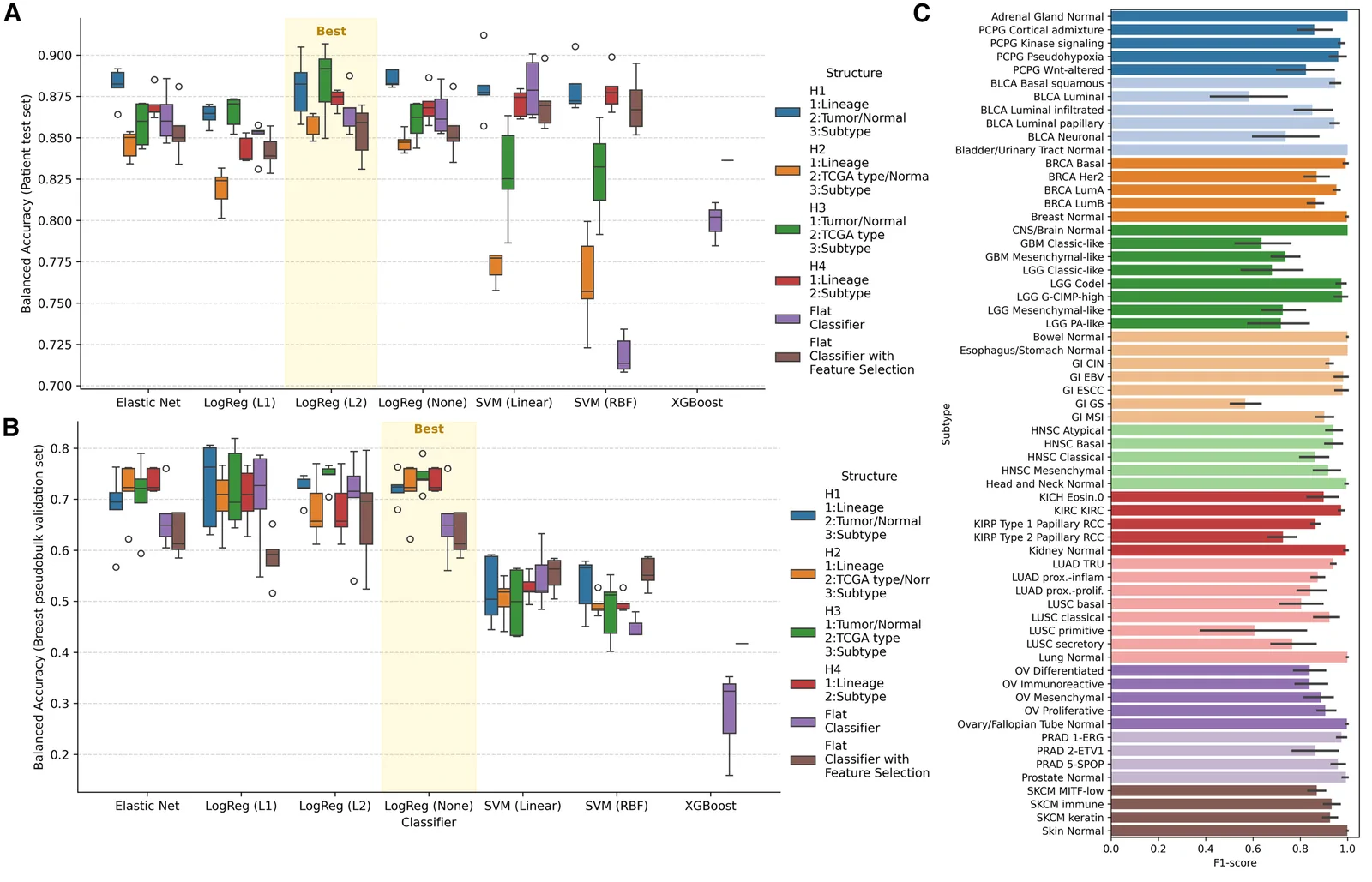
Benchmarking results of HC systems for cancer subtype matching (A) Balanced accuracy of different inner classifier and hierarchy combinations and their flat classifier counterparts with and without MRMR-based feature selection in patient data (CV = 5). Best average performing model highlighted. Boxes indicate the interquartile range (IQR), and whiskers represent the full distribution excluding outliers (o). (B) Balanced accuracy of the same models on external breast cancer pseudobulk set (CV = 5). Best average performing model highlighted. Boxes indicate the IQR, and whiskers represent the full distribution excluding outliers (o). (C) F1-score on patient samples for each class on test folds (CV = 5) with the HC model using logistic L2 inner classifiers (hierarchy 3: tumor/normal, TCGA type, and subtype) (error bars = 95% confidence interval).

Figure 2 summarizes the performance of the evaluated HC designs and inner classifiers under CV. Across test folds, balanced accuracy varied depending on classifier-hierarchy combinations. LogReg-based hierarchical models performed most consistently, achieving balanced accuracy up to ∼0.89, whereas SVM-based classifiers showed more variable performance, particularly in hierarchies incorporating TCGA type labels. Among flat models, linear SVM achieved the strongest performance with balanced accuracy approaching 0.88 (Figure 2A). Among the hierarchy structures, H3 (tumor/normal, TCGA type, and subtype) was the best overall performer across models and outperformed all comparative flat approaches. H1 also provided competitive performance whereas H2 consistently failed to outperform flat classifiers. Even the simplified two-level structure in H4 (organ and subtype) improved performance for most classifier types, demonstrating that incorporating even minimal biological hierarchy provides measurable benefit.

Preservation of subtype-specific signals in breast cancer pseudobulk samples was seen as clustering of patient tissue samples by subtype after batch correction (Figure S4). Performance differences became more pronounced on this external validation set. The best flat model (LogReg with L1 penalty) achieved 0.70 balanced accuracy, whereas the best hierarchical model reached 0.75. LogReg benefited most from hierarchical modeling, with all hierarchical logistic models outperforming their flat counterparts (Figure 2B).

Integrating performance across datasets, the best model overall was LogReg with L2 penalty under hierarchy H3, achieving 0.88 balanced accuracy on patient samples and 0.75 on pseudobulk data. This model classified normal samples and common subtypes with near-perfect accuracy. Rarer or less well-defined subtypes, such as neuronal and luminal bladder cancer, classic- or mesenchymal-like glioblastoma, pilocytic astrocytoma–like (PA-like) lower-grade glioma, genomic stability (GS)-type GI cancers, and primitive/secretory lung squamous carcinoma, were more challenging (Figure 2C). Critically, misclassifications remained within the correct tissue lineage, demonstrating the biological plausibility enforced by the hierarchical approach (Figure S5). In the external GEO: GSE26712 OV dataset, this same HC approach achieved 0.80 balanced accuracy compared to 0.76 for the flat counterpart.

Although the performance gains achieved by HC were generally modest, even small improvements are meaningful given the difficulty of the multiclass subtype prediction task and the already strong performance of the best flat models. Moreover, the primary advantage of the HC framework lies in its improved interpretability, as predictions can be traced through biologically meaningful decision nodes and linked to tumor- and subtype-specific molecular features. Additionally, the larger performance gains observed on the independent pseudobulk and bulk OV datasets suggests improved generalization, which is particularly important for transferring subtype labels between domains. Together, these results indicate that HC can provide modest improvements in predictive performance while offering more biologically interpretable subtype predictions than flat approaches.

### Hierarchy-aware feature selection uncovers biologically informative and subtype-specific markers

The added value of the implemented feature selection process, particularly from a biological perspective, was evaluated by assessing whether the selected features remain stable across CV folds. Following the initial trimming of 80% of features based on mutual information scores calculated on the patient dataset, maximum relevance minimum redundancy (MRMR) was applied to select features for each inner classifier. The resulting gene sets provide insight into classifier decision-making and highlight subtype-specific or broadly informative biomarkers.

**Figure 3:**
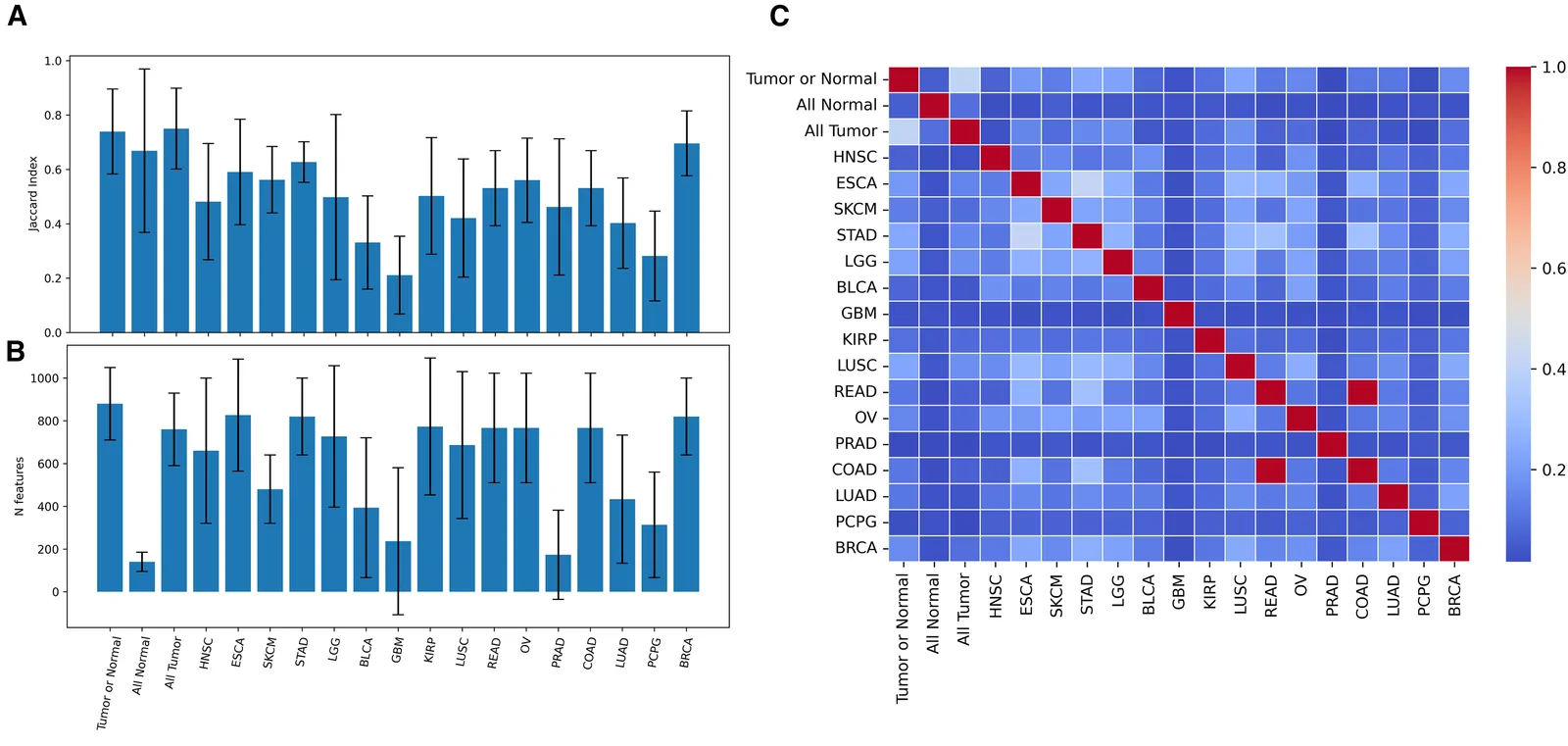
Robustness and number of selected features across folds and their node specificity (A) Robustness of the selected features per node via the Jaccard index across folds in best hierarchical model (CV = 5). Error bars represent standard deviation. (B) The number of features selected for each node in the hierarchy. Error bars represent standard deviation. (C) Jaccard index between sets of selected features selected between different nodes in hierarchy 3.

The stability and specificity of the selected feature sets were evaluated for the best-performing hierarchical model (Figure 3). Feature stability across CV folds was highest for classifiers located at the top of the hierarchy, including those distinguishing tumors from normal samples, separating normal tissues by organ, and classifying TCGA cancer types. Among subtype classifiers, breast invasive carcinoma (BRCA), stomach adenocarcinoma (STAD), and esophageal carcinoma (ESCA) showed the most consistent feature sets, whereas glioblastoma multiforme (GBM), bladder urothelial carcinoma (BLCA), and pheochromocytoma and paraganglioma (PCPG) displayed lower stability (Figure 3A). The number of selected features varied across classification tasks. The tumor-normal classifier and subtype classifiers for ESCA, STAD, and BRCA required the largest feature sets (>800 features on average), while fewer features were needed for separating normal tissues and for GBM and prostate adenocarcinoma (PRAD) subtype classification (Figure 3B). Specificity of the selected features for each classification task was shown to be relatively high. Most inner classifiers selected features that were not highly similar to any of the other inner classifiers within the hierarchy with the exception of rectum adenocarcinoma (READ) and colon adenocarcinoma (COAD), which shared the same subtype labels. Otherwise, the most similar sets of features were often present in biologically similar cancers or tissue types such as STAD and ESCA. PRAD, PCPG, and GBM tended to have highly unique features compared to any of the other cancer types (Figure 3C).

Despite the large pool of retained features after the initial trimming step, each inner classifier relied on a substantially smaller subset, selecting on average 546 genes. In some folds, accurate predictions were achieved with as few as 10 features, demonstrating that only a compact set of informative genes is required for certain classification tasks. More complex subtasks deeper in the hierarchy occasionally required the maximum number of features (1000), reflecting the increased difficulty of fine-grained subtype discrimination. Most predictive power was concentrated within a relatively small subset of features, with additional features contributing only limited performance gains. This suggests that much of the predictive signal is concentrated within a relatively small subset of genes, although additional features still provided modest gains for several nodes, indicating a contribution from more diffuse transcriptional programs (Figure S6).

The biological relevance of the selected features was assessed by examining the coefficients of LogReg models and the log fold changes of selected genes between subtypes in both CLs and patient samples (Figures S7 and S8). Clear subtype-specific expression patterns were observed in the patient tumors, whereas the corresponding CLs often captured only a subset of these transcriptional differences. The degree of concordance varied across cancer types, highlighting where *in vitro* models reflect or fail to reproduce patient-level subtype variation.

Across the nodes, the classifiers exhibited clear subtype-specific weighting patterns, with several genes showing strong and distinct coefficient signatures. The coefficient distributions showed that some classifiers relied on a narrow set of large-magnitude coefficients, whereas others displayed more diffuse weighting patterns, indicating varying degrees of subtype separability across cancers. Many coefficients were close to zero, indicating limited individual predictive contribution of certain selected genes. Regularization can mitigate the influence of such features by shrinking their coefficients, which may explain why regularized LogReg models occasionally outperformed their non-regularized counterparts (Figure S8).

Preservation of gene expression patterns between patient tumors and CLs was further assessed by comparing gene-wise *Z* scores across both domains using the predicted subtype labels for the CL dataset. Across cancer types, the combined *Z* scores of genes showed strong overall correlation between patient tissues and corresponding CLs, indicating that the relative expression specificity of most genes is reasonably well retained *in vitro*. Correlations improved further when restricting the comparison to only those genes selected by MRMR for each node. However, kidney renal papillary cell carcinoma (KIRP), brain lower grade glioma (LGG), LUSC, and especially lung adenocarcinoma (LUAD), saw a decrease in correlation using the selected gene sets (Figure S9). For LUAD this was especially apparent for the TRU subtype, which was very lowly correlated between patients and CLs due to only one CL being assigned to the subtype (originally labeled as a COAD CL) (Figure S10). This is expected as the features these classifiers have selected are not meant to separate different TCGA types, but different subtypes within a single cancer. When taking this into account and exploring the subtype level, only 4 subtypes did not see an improvement in their alignments between domains using the sets of genes selected by the model.

Furthermore, functional enrichment analysis was performed to characterize the biological processes associated with the node-specific gene sets (see STAR Methods for details). After enrichment analysis, significant pathways (*p* < 0.05) were retained and grouped into broader biological themes to reduce redundancy. For each tumor, the dominant theme was defined based on the most significant pathway. Genes contributing to the enriched pathways within this theme were aggregated to identify key genes, and putative biomarkers were defined as those most frequently appearing across the enriched pathways, representing core components of the tumor-associated transcriptional program. Notably, as the HC model was trained on patient tumors and subsequently used to assign subtype labels to CLs, the biological characterization of the selected features provides an additional layer of validation for the annotation framework. These analyses help assess the biological plausibility of the transferred labels and provide mechanistic support that the annotated CLs represent the corresponding patient-derived molecular subtypes. In addition, the identified biomarkers and pathways may help guide the selection of CLs that best capture specific biological processes for downstream experimental and translational studies. Table 1 summarizes the main biological pathways enriched for the genes selected across tumor types and highlights the genes most frequently contributing to these pathways, reported as candidate biomarkers for each tumor-specific theme. Analysis of top genes selected by the best hierarchical model is in Table S2.

**Table 1:** Summary of enriched biological pathways and candidate biomarkers across tumor types

Tumor	Dominant biological theme	Representative pathway	Source	Best *p* value	Putative biomarker (correlation)	Biological interpretation
BLCA	development/differentiation	anatomical structure morphogenesis	GO:BP	7.86E−09	HEY1 (0.81), TBX3 (0.69), SPHK1 (0.20)	reflects developmental or tissue differentiation programs
BRCA	cell cycle/mitotic division	cell cycle checkpoints	REAC	9.32E−07	CDC20 (0.85), AURKB (0.86), SGO1 (0.78)	indicates proliferative activity and regulation of mitosis and chromosome segregation
COAD	other biological process	GABA synthesis, release, reuptake, and degradation	REAC	0.001514	RAB3A (0.58), SYT1 (0.65), CPLX1 (0.47)	represents the dominant enriched biological program for this tumor gene set
ESCA	development/differentiation	epidermis development	GO:BP	4.57E−07	SLC44A4 (0.81), CSTA (0.86), TMEM79 (0.48)	reflects developmental or tissue differentiation programs
HNSC	extracellular matrix/stromal remodeling	collagen metabolic process	GO:BP	8.09E−08	COL1A1 (0.74), EMILIN1 (0.48), COL5A1 (0.22)	suggests extracellular matrix remodeling, stromal interaction, or invasive behavior
KIRP	other biological process	glycosphingolipid biosynthesis - lacto and neolacto series	KEGG	0.012685	B4GALNT2 (0.54), A4GALT (0.86), B3GNT4 (0.53)	represents the dominant enriched biological program for this tumor gene set
LGG	development/differentiation	embryonic skeletal system morphogenesis	GO:BP	0.006551	SHOX2 (0.57), HOXA5 (0.55), HOXB3 (0.58)	reflects developmental or tissue differentiation programs
LUAD	cell cycle/mitotic division	nuclear chromosome segregation	GO:BP	5.51E−12	NUF2 (0.88), BIRC5 (0.86), NEK2 (0.86)	indicates proliferative activity and regulation of mitosis and chromosome segregation
LUSC	development/differentiation	developmental process	GO:BP	4.14E−06	FOXA1 (0.85), HES6 (0.82), TRIM16 (0.88)	reflects developmental or tissue differentiation programs
OV	other biological process	integrin cell surface interactions	REAC	5.78E−10	COL5A2 (0.31), COL5A1 (0.22), COL1A1 (0.74)	represents the dominant enriched biological program for this tumor gene set
PCPG	other biological process	small molecule catabolic process	GO:BP	0.006745	SULT2A1 (0.76), APOE (0.85), SCARB1 (0.71)	represents the dominant enriched biological program for this tumor gene set
PRAD	other biological process	vascular smooth muscle contraction	KEGG	0.003345	KCNMB3 (0.59), CALM2 (0.83), ITPR3 (0.82)	represents the dominant enriched biological program for this tumor gene set
READ	other biological process	GABA synthesis, release, reuptake, and degradation	REAC	0.001514	RAB3A (0.58), SYT1 (0.65), CPLX1 (0.47)	represents the dominant enriched biological program for this tumor gene set
SKCM	immune/inflammatory signaling	cytokine signaling in immune system	REAC	0.002494	IRF1 (0.33), IL2RG (0.71), TNFRSF14 (0.88)	suggests immune-related or inflammatory transcriptional programs
STAD	other biological process	GABA synthesis, release, reuptake, and degradation	REAC	0.000651	RAB3A (0.58), SYT1 (0.65), CPLX1 (0.47)	represents the dominant enriched biological program for this tumor gene set
All tumor	protein synthesis/translation	eukaryotic translation elongation	REAC	0.000121	RPS10 (0.94), RPL36A (0.85), RPL26 (0.93)	reflects elevated translational activity and ribosome-associated processes

For each tumor, the table reports the dominant biological theme identified by enrichment analysis, the most significant representative pathway and its source database (Gene Ontology Biological Process [GO:BP], Reactome [REAC], and Kyoto Encyclopedia of Genes and Genomes [KEGG]), the corresponding *p* value, and the genes most frequently contributing to the enriched pathways, reported as putative biomarkers together with a brief biological interpretation. Putative biomarkers are accompanied with Pearson correlation of *Z* scores between patients and CLs across subtypes.

### Prediction reliability metrics highlight complementary sources of uncertainty and reveal potential mislabeling and gaps in cancer CL representation

The best-performing hierarchical model (LogReg with L2 regularization under hierarchy H3) was applied to CCLE and GDSC transcriptomic profiles to predict lineage, cancer type, and molecular subtype. Prediction reliability was assessed using three complementary metrics: class probability entropy, top-two probability difference, and AD distance ratio computed using the top 20% of mutual information-filtered genes. These metrics were evaluated using an external breast cancer pseudobulk dataset and subsequently applied to CL predictions.

On the external pseudobulk dataset, AD distance showed minimal correlation with misclassification rates for hierarchical models (r = 0.01) but was substantially more informative for flat SVM classifiers (r = 0.48), indicating greater sensitivity of flat models to domain similarity. Entropy and top-two probability difference were highly correlated across CL predictions (Pearson r = 0.96; Spearman r = 0.91), whereas neither metric correlated meaningfully with AD distance, highlighting their distinct roles in capturing probabilistic uncertainty and domain shift, respectively.

Highest confidence was observed for bladder CL predictions, followed by bowel, prostate, and kidney CLs. Prostate and lung CLs exhibited the largest AD distances, whereas skin and bowel CLs were closest to the training domain. Metastatic CLs showed increased probabilistic uncertainty relative to primary CLs despite comparable AD distances, suggesting retention of global tissue similarity but reduced subtype specific signal resolution. In total, 612 CL predictions were labeled high confidence, 141 as medium confidence, and 49 as low confidence. High-confidence predictions are recommended for direct downstream use, medium-confidence predictions should be interpreted cautiously, and low-confidence predictions should be treated as ambiguous. Together, these results indicate that combining probabilistic confidence metrics with AD distance enables robust identification of unreliable or out-of-domain predictions.

Subtype predictions revealed various biologically plausible annotation mismatches. Overall, 94% of CLs were assigned to their originally annotated tissue lineage. Three nominally non-cancerous CLs (HMEL, BPH-1, and PWR-1E) were classified as cancerous and reassigned to alternative lineages. 43 of 802 CL profiles (5.4%) were reassigned to a new lineage, most commonly involving reassignment to the bladder, GI, skin, or lungs. Misclassifications were largely restricted to related epithelial lineages, consistent with shared transcriptional programs.

While most CLs were assigned to their expected cancer types and subtypes, several notable cross-lineage predictions were observed (Data S1). Bladder, breast, and brain cancer models revealed both subtype-specific biases and examples of CLs whose transcriptional profiles more closely resembled other tumor types. Lung cancer exhibited the highest degree of discordance, with numerous non-small-cell lung cancer CLs aligning with GI, breast, kidney, or bladder cancer subtypes, suggesting shared epithelial programs, squamous differentiation, or potential annotation issues. Similar cross-lineage assignments were observed among GI, head and neck, kidney, and OV CLs, although some discrepancies were dataset-dependent and differed between CCLE and GDSC expression profiles, highlighting the impact of data source on CL characterization. In contrast, all skin cancer CLs were correctly assigned to their nominal lineage, while several CLs originating from other tissues, particularly glioma models, were predicted as melanoma, including LN464, which has previously been reported as a problematic and likely misidentified melanoma CL.^16^ Collectively, these findings demonstrate that although most CLs retain lineage-specific transcriptional characteristics, a subset displays molecular profiles that are inconsistent with their annotated tissue of origin, reflecting either biological convergence across cancers, genetic drift, or annotation and identity issues.

To further corroborate the CL lineage predictions, the results were compared with entries in the International Cell Line Authentication Committee (ICLAC) Register of Misidentified Cell Lines (Version 14).^3^ Fourteen CLs included in the study were present in the registry. Among these, GT3TKB originally established as a stomach cancer CL but subsequently reassigned to lung cancer, was predicted as lung cancer by the HC. Likewise, ONCO-DG-1, originally annotated as papillary thyroid carcinoma and later reassigned to ovarian carcinoma, was predicted as OV. LN464 being melanoma was also corroborated by ICLAC. For contaminated CLs with uncertain or unresolved origins, MEL-HO was predicted as melanoma, NCI-H630 as a GI cancer, and OC 314 and OC 316 as OV. GOS-3 was originally annotated as astro-oligodendroglioma but has been reassigned to glioblastoma. This CL was predicted as LGG by the HC model. The remaining six ICLAC-listed CLs showed no lineage discrepancy between their reported annotation and ICLAC records, and their predicted cancer types were consistent with these annotations. Thus, known lineage reassignments recorded in the ICLAC registry were recapitulated by the HC, while plausible lineage assignments were provided for CLs with unresolved origins.

Comparison of predictions between CCLE and GDSC profiles for 62 shared CLs showed high concordance, with consistent TCGA cancer type predictions for 55 CLs and matching subtype predictions for 45 CLs. Only one CL (A427) was consistently assigned to a different tissue lineage across both datasets, suggesting substantial divergence from its nominal annotation. In these cases, the predictions of higher confidence should be preferred.

**Figure 4:**
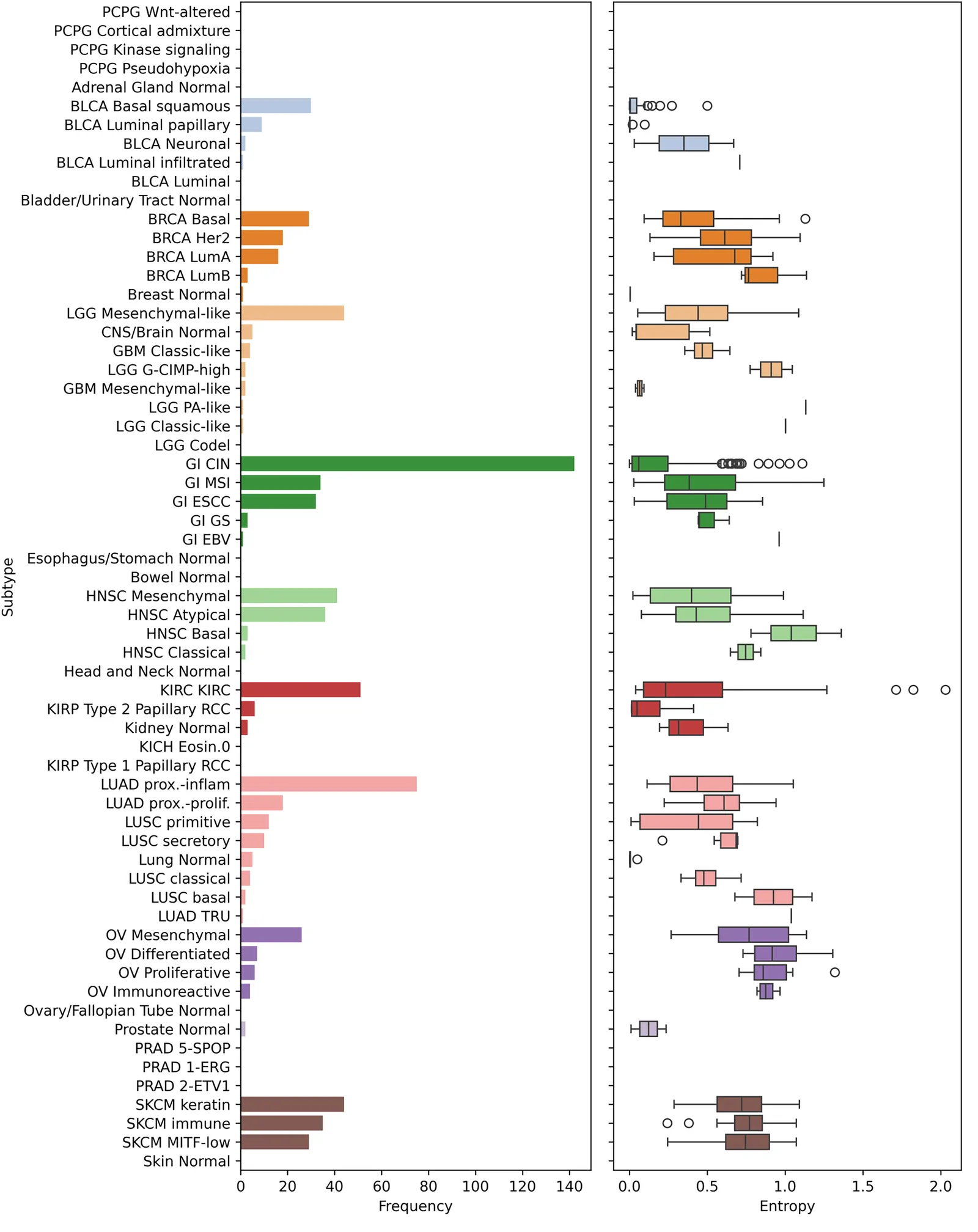
The frequency of CLs predicted for each subtype and the associated probability distribution entropies per subtype Colors indicate the different lineages. Results for HC model with inner LogReg with L2 penalty and hierarchy separating tumor samples from normal samples, then TCGA types and finally subtypes (H3). Lower entropies imply more confident predictions. Boxes show median, 25th, and 75th percentiles.

Several clinically relevant tumor subtypes were found to be underrepresented or absent among CLs (Figure 4). Immune-rich subtypes, including BLCA luminal infiltrated^17^ and immune-defined kidney subtypes (kidney chromophobe [KICH] Eosin.0 and KIRP type 1),^18^^,^^19^ were rarely or never assigned, likely reflecting the absence of immune components in CL models. Instead, KIRP type 2, associated with worse prognosis and clearer molecular markers,^19^ was more frequent. In lung cancer, the well-differentiated LUAD TRU subtype was rarely represented, whereas aggressive proximal inflammatory and proliferative subtypes predominated, as reported in other studies.^20^ Overall, CLs were enriched for aggressive, basal-like transcriptional programs, while more differentiated or immune-dependent subtypes were underrepresented (Figure 4A).

Overall, the most common subtype in CLs was predicted to be chromosomally instable GI cancer, the most common subtype in GI cancer patients. However, as the subtype is broadly characterized by chromosomal instability, this subtype may capture many unreliably predicted subtypes. This can be seen in the low probability entropies in many of these predictions (Figure 4B). These findings indicate that prediction confidence metrics can identify unreliable annotations and that existing CL panels systematically favor aggressive tumor phenotypes. This bias has important implications for preclinical modeling, drug response interpretation, and biomarker discovery, underscoring the need for improved representation of tumor heterogeneity in CL resources. Complete CL annotations and metrics are provided in Data S1.

Yu et al.^13^ assigned CCLE CLs to molecular subtypes across nine tumor types, five of which overlapped with the subtype definitions in this study (BRCA, LUAD, LUSC, HNSC, and skin cutaneous melanoma [SKCM]) enabling direct comparison. Among 215 CL profiles, 134 (62.3%) received the same subtype assignment. Most discrepancies occurred between subtypes within the same tumor type or between LUAD and LUSC. These differences likely reflect methodological distinctions. Yu et al. performed subtype classification independently within each tumor type using the nearest template prediction approach and did not include normal tissue samples. Consequently, their framework could not assign CLs to alternative tumor types or to normal tissues. In contrast, the presented hierarchical classifier was trained across a range of tumor types and included normal samples, allowing reassignment when a CL’s transcriptional profile more closely matched another tumor type or normal tissue. This design enables identification of broader transcriptional programs across tumors while accounting for potential misannotation or divergent lineage identity in established CLs. Furthermore, the hierarchical structure improves interpretability by allowing predictions to be traced through biologically meaningful lineage and subtype decisions.

## Discussion

The described HC approach significantly improves the alignment of cancer CLs with patient tumor subtypes, achieving balanced accuracy of up to 0.89. This approach provides a robust, interpretable foundation for improving experimental model selection and translational research. By leveraging hierarchical modeling and task-specific feature selection, the analysis recapitulated known biological biases and misannotations and uncovered critical gaps in current CL panels—specifically the absence of low-aggressiveness and high-immune infiltration subtypes like BLCA Luminal and LGG Codel.

These findings are consistent with previous studies demonstrating that the transcriptional similarity between tumors and CLs varies substantially across cancer types and that many commonly used CLs incompletely capture the molecular heterogeneity observed in patient tumors.^6^ Importantly, the identification of tumor subtypes lacking representative CLs highlights a persistent limitation of conventional *in vitro* cancer models. Previous studies have emphasized the value of patient-derived primary cultures as complementary preclinical models capable of capturing clinically relevant tumor characteristics that are often absent in established CLs.^21^ The development and integration of such patient-derived models may therefore help address the identified subtype representation gaps, especially in precision oncology, where accurate preclinical models are essential for translating molecular findings into clinically actionable therapeutic strategies.^22^

### Limitations of the study

Despite these advances, limitations regarding batch effects and metastatic data remain. Specifically, the batch correction utilized covariates with tissue-of-origin and tumor status, which may have introduced mild transductive effects, or masked real tumor programs through over-correction, though performance on uncorrected TCGA data remained similar. Additionally, prediction sensitivity may have been limited for rarer subtypes with smaller sample sizes, even with the applied 20-sample threshold. Tumor purity, the absence of metastatic tumor samples during training, and the lack of microenvironmental components in CLs may have further constrained alignment accuracy, as many subtype definitions partially depend on immune or stromal signals that are not represented *in vitro*. Consequently, missing subtype assignments in CLs may reflect technical limitations, sampling effects, or the absence of tumor microenvironmental signals rather than a true absence of the corresponding subtype biology.

## Resource availability

### Lead contact

Requests for further information and resources should be directed to and will be fulfilled by the lead contact, Vittorio Fortino (vittorio.fortino@uef.fi).

### Materials availability

This study did not generate new unique reagents.

### Data and code availability

• Processed data are publicly available at Zenodo [https://doi.org/10.5281/zenodo.21531844] as of the date of publication
• All original code is available at https://github.com/UEFBiomedicalInformaticsLab/HiSACS
• Any additional information required to reanalyze the data reported in this paper is available from the lead contact upon request.

## Acknowledgments

This work was supported by the 10.13039/501100004012Jane and Aatos Erkko Foundation [210026 to V.F.]; 10.13039/501100006306Sigrid Jusélius Foundation; and the 10.13039/501100008413Instrumentarium Science Foundation [250019 to J.M.]. The computational analyses were performed on servers provided by the Bioinformatics Center of the 10.13039/100007753University of Eastern Finland. The graphical abstract was created using BioRender.

## Author contributions

J.M., writing – review and editing, writing – original draft, visualization, software, methodology, investigation, formal analysis, data curation, and conceptualization; T.J.R., methodology and writing – review and editing; V.F., supervision, project administration, writing – review and editing, validation, methodology, conceptualization, and funding acquisition.

## Declaration of interests

The authors declare no competing interests.

## STAR★Methods

### Key resources table

**Table:** 

REAGENT or RESOURCE	SOURCE	IDENTIFIER
**Deposited data**		

Scripts and code	This paper	https://github.com/UEFBiomedicalInformaticsLab/HiSACS
Processed data	This paper	https://doi.org/10.5281/zenodo.21531844
Primary tumor and normal tissue RNA-seq data and subtype annotations	TCGAbiolinks (v2.25.3)	https://doi.org/10.18129/B9.bioc.TCGAbiolinks
Normal RNA-seq profiles	GTEx (v8)	https://doi.org/10.1038/ng.2653
CCLE cell line RNA-seq profiles	DepMap portal (22Q3/23Q2 release)	https://doi.org/10.6084/m9.figshare.22765112
GDSC cell line RNA-seq profiles	Expression Atlas	https://doi.org/10.1093/nar/gks1111
Single-cell RNA-seq data from breast cancer	CellxGene	GEO: GSE176078; https://www.ncbi.nlm.nih.gov/geo/query/acc.cgi?acc=GSE176078
Ovarian cancer microarray dataset	Bonome et al.^23^	GEO: GSE26712; https://www.ncbi.nlm.nih.gov/geo/query/acc.cgi?acc=GSE26712
Ovarian cancer microarray dataset subtype annotations	Konecny et al.^24^	https://doi.org/10.1093/jnci/dju249

**Software and algorithms**		

R v4.2.2	R Core Team	N/A
Python v3.9.12	Python Software Foundation	N/A
pyComBat v0.3.3	Behdenna et al.^25^	https://github.com/epigenelabs/pyComBat
modified sklearn-hierarchical-classification v1.3.2	globality-corp	https://github.com/globality-corp/sklearn-hierarchical-classification
scikit-learn v1.5.2	Pedregosa et al.^26^	https://pypi.org/project/scikit-learn/1.5.2/
applicability-domain v0.1.0	yu9824	https://github.com/yu9824/applicability_domain/
mrmr-selection v0.2.8	Samuele Mazzanti	https://github.com/smazzanti/mrmr
pandas v2.2.3	The pandas development team https://doi.org/10.5281/zenodo.3509134	https://pypi.org/project/pandas/2.3.2/
numpy v2.0.2	Harris et al.^27^	https://pypi.org/project/numpy/2.0.2/
matplotlib v3.9.2	Hunter^28^	https://pypi.org/project/matplotlib/3.9.2/
seaborn v0.13.2	Waskom^29^	https://pypi.org/project/seaborn/
umap-learn v0.5.6	McInnes et al.^30^	https://pypi.org/project/umap-learn/
scanpy v1.10.3	Wolf et al.^31^	https://pypi.org/project/scanpy/1.10.3/
g:Profiler	Kolberg et al.^32^	https://biit.cs.ut.ee/gprofiler
xgboost v2.1.1	N/A	https://pypi.org/project/xgboost/2.1.1/

### Method details

#### Data collection and processing

Patient datasets (TCGA, GTEx) were used for model training and cross-validation (CV), while CL datasets (CCLE, GDSC) were used as prediction targets. A pseudobulk breast cancer dataset (CellxGene) was used for external evaluation of model performance along a bulk ovarian cancer dataset (Gene Expression Omnibus).

Primary tumor and adjacent normal tissue RNA-seq data (TPM) and subtype annotations were obtained from TCGA using TCGAbiolinks (v2.25.3) in R (v4.2.2). Additional non-cancerous tissue samples were downloaded from GTEx v8.^33^ CCLE TPM expression data and metadata were obtained from the DepMap portal (22Q3/23Q2 release), while GDSC expression data were retrieved via the Expression Atlas.^34^^,^^35^ Single-cell RNA-seq data were obtained from GEO: GSE176078 via CellxGene.^36^^,^^37^ All datasets were mapped to the human reference genome (GRCh38), and gene identifiers were standardized to HGNC gene symbols using limma.^38^ Sample inclusion criteria are detailed in Table S1.

For GDSC, duplicate gene measurements were averaged, genes missing values in >100 samples were removed, and remaining missing values were imputed using the median. TCGA READ and COAD were grouped as bowel cancer, and ESCA and STAD were grouped as esophagus/stomach based on molecular similarity. All gastrointestinal (GI) cancers shared subtype definitions due to overlapping genetic patterns.^39^ Metadata were manually harmonized across datasets, and undefined subtypes and the 10 subtypes represented by fewer than 20 patient samples were excluded to only target robust major programs.

Gene expression values were log2-transformed, and only genes shared across all datasets (*n* = 13,007) were retained. Single-cell data were aggregated into pseudobulk samples per patient, generating separate tumor and non-cancerous pseudobulk profiles, which were converted to TPM and processed identically to bulk samples. Batch correction was performed using pyComBat (v0.3.3), with dataset source as the batch variable and organ of origin and tumor status (Tumor / Normal) included as covariates.

External validation was performed using the dataset GEO: GSE26712.^23^ This dataset comprised 185 ovarian cancer samples and 10 healthy ovary samples. The ovarian cancer cohort included samples representing all four molecular subtypes of high-grade serous ovarian cancer.^24^ To improve the comparability of gene expression measurements between the RNA-seq and microarray data, the 8,678 shared gene expression values were standardized using z-score normalization prior to batch correction.

#### Cancer CL annotation and subtype selection

TCGA transcriptomic subtypes and matched GTEx tissues were curated to support biologically grounded modeling of tumor subtypes in CLs. Subtyping selection from those contained in TCGAbiolinks was guided by clinical relevance and robustness, sufficient sample size for reliable model training, and compatibility between tumor-intrinsic transcriptional programs and those retained in CLs. The selected subtypes capture major axes of tumor biology, including immune infiltration, chromosomal instability, epigenetic alterations (e.g., G-CIMP), and differentiation state, and encompass established classification frameworks such as BRCA PAM50, ovarian HGSC subtypes and LUAD/LUSC expression programs (Table S1). The goal was to select subtype labelling schemas that are commonly used, biologically relevant and detectable through mRNA.

CLs were mapped to patient cohorts through a stepwise filtering process. First, CLs were restricted to those whose OncoTree lineage matched the corresponding TCGA and GTEx tissues; OncoTree provides standardized cancer lineage and disease annotations across tumor types.^40^ Second, CLs were filtered by primary disease to retain only those corresponding to TCGA cancer types. Third, CLs representing subtypes not defined within TCGA classification schemes were excluded. Finally, CLs lacking sufficient metadata for lineage or disease assignment were removed.

#### Evaluation of batch correction performance

Alignment between CLs and patient tumors was quantified using the tissue match fraction based on each CL’s ten nearest neighboring patient samples in PCA space. PCA was performed on the integrated gene expression matrix, and the top 20 components were retained. The tissue match fraction was defined as the proportion of nearest neighbors sharing the CL’s tissue lineage. Standardized gene expression differences were measured using Cohen’s d. For each gene, Cohen’s d was computed as the mean expression difference between CLs and patient samples divided by the pooled standard deviation. Effect sizes were calculated before and after batch correction to assess reduction of systematic dataset-specific differences. Finally, a simple logistic regression (LogReg) classifier was trained to see if it was able to separate samples between patients and CLs after correction.

To assess the impact of batch correction and normal sample inclusion on prediction performance, an ablation study was conducted. Models were trained and evaluated using the best-performing inner classifier across all hierarchies (LogReg L2) under multiple conditions: with and without ComBat batch correction, with and without covariate adjustment, and with normal samples included or excluded. Furthermore, an alternative batch-correction strategy based on linear regression was investigated. Analogous to batch correction in limma, this approach corrected batch effects while preserving biological variation associated with Oncotree lineage and Tumor/Normal status.^38^

#### HC–based strategies for matching cancer CLs to tumor subtypes

Classification was performed using a HC framework that progressively refines predictions from broad biological categories to tumor subtypes. HC models were implemented using the sklearn-hierarchical-classification package (v1.3.2), which was extended to support node-specific feature selection and coefficient extraction from internal classifiers. Four alternative hierarchies were designed to capture distinct biological and clinical assumptions. Hierarchy 1 (H1: Organ, Tumor/Normal, Subtype) emphasizes tissue of origin by sequentially assigning organ lineage, tumor status, and subtype. Hierarchy 2 (H2: Organ, TCGA Cancer Type/Normal, Subtype) introduces TCGA types as an intermediate resolution for tumor samples. Hierarchy 3 (H3: Tumor/Normal, TCGA Cancer Type, Subtype) prioritizes early separation of tumor and normal samples prior to cancer type and subtype assignment. Hierarchy 4 (H4: Organ, Subtype) provides a simplified two-level structure, enabling evaluation of performance in the absence of intermediate decision nodes. Visual representations of all hierarchies are provided in Figure S1.

Each parent node in the hierarchy was associated with a multi-class classifier responsible for assigning samples to its child nodes, following a “classifier-per-parent-node” strategy, which offers a favorable balance between accuracy and computational efficiency relative to alternative hierarchical formulations.^7^ Multiple classification algorithms were evaluated, including linear and RBF support vector machines (SVMs), LogReg with L1, L2, or no regularization, and Elastic Net. All models were additionally trained in a flat (non-hierarchical) setting for baseline comparison, with XGBoost included as an additional flat benchmark.

Hyperparameters were optimized using grid search with nested five-fold CV, where inner folds were drawn exclusively from training splits of the outer loop to prevent data leakage. Regularization strengths (C = 0.01, 0.1, 1, 10, 100) were tested for penalized models, and Elastic Net mixing parameters (l1_ratio = 0.25, 0.5, 0.75) were evaluated. Balanced accuracy (mean class recall) was used as the primary tuning and evaluation metric to account for class imbalance, and class-specific performance was summarized using F1 scores.

#### Feature selection and CV framework

Feature selection was integrated into the HC pipeline to improve generalization and interpretability. First, the bottom 80% of features were removed via mutual information (MI) scores between gene expression and subtypes across TCGA and GTEx datasets—a threshold determined to minimize performance loss while reducing cost. Subsequently, the Maximum Relevance Minimum Redundancy (MRMR) framework was applied within each hierarchy, using F-statistic for relevance and Pearson correlation for redundancy. The optimal number of features was determined through nested 5-fold CV, during which feature selection and hyperparameter tuning were jointly optimized. Candidate feature set sizes included 10, 20, 40, 80, 160, 320, 640, and 1000, balancing model granularity with complexity.

Primary tissue samples from TCGA and GTEx were partitioned into five stratified folds while preserving subtype distributions. For each fold, MI filtering, HC model training, and evaluation were conducted. Inner classifiers underwent nested CV to identify the optimal hyperparameters and feature subsets, after which final models were trained on the complete dataset using the selected configurations.

#### Feature selection post-hoc analysis

Log2 fold-change (log2FC) analysis was performed for patient tumor samples and corresponding CLs. For each TCGA cancer type, the gene set was derived from the feature selection stage of the best-performing predictive model. Expression data were grouped by TCGA subtype annotations for patient samples and by predicted subtypes for CLs. For each cancer type and subtype, log2FC values were calculated by comparing the mean expression of subtype samples to the mean expression of all other subtypes within the same cancer type. In the CL dataset, subtypes represented by fewer than two samples and cancer types with fewer than two subtypes were excluded.

Gene expression specificity across classes was further evaluated by averaging samples by cancer type and subtype and computing gene-wise z-scores separately for patient and CL datasets, using predicted subtypes for CLs. Z-scores were combined across groups using Stouffer’s method,^41^ and Pearson correlation between combined scores was used to assess cross-domain consistency of gene markers.

To assess gene contributions to subtype discrimination, coefficients from the best hierarchical classification model were extracted for each decision node. For each class, the three genes with the largest absolute coefficients were identified, and the union of these genes was used to construct a coefficient matrix displaying subtype-specific and shared contributions. In binary nodes, coefficients for the opposing class were represented by negative values.

#### Pathway enrichment analysis and identification of tumor-associated biological pathways

Functional enrichment analysis was used to characterize biological processes associated with tumor-specific gene sets. For each TCGA tumor type, the top 100 genes were analyzed using g:Profiler (Homo sapiens) against the Gene Ontology Biological Process (GO:BP), Reactome, and Kyoto Encyclopedia of Genes and Genomes (KEGG) databases.^32^ Over-representation analysis was performed, and pathways with *p* < 0.05 were considered significant. For each tumor type, the 30 most significant pathways were retained, and genes contributing to each enrichment were extracted.

Enriched pathways were grouped into broader biological themes using rule-based keyword mapping. Themes included protein synthesis and translation; cell cycle and mitotic division; extracellular matrix organization and stromal remodeling; immune and inflammatory signaling; development and differentiation; DNA replication and repair; metabolic reprogramming; and neural signaling and differentiation. For each tumor type, the dominant biological theme was defined as the theme containing the pathway with the lowest enrichment *p*-value and the highest supporting gene overlap. The most significant pathway within this theme was reported as the representative pathway, and genes contributing to enrichment across associated pathways were aggregated.

Key enriched genes were defined as the union of genes supporting the dominant theme. Putative biomarker genes were further identified by ranking genes according to their recurrence across enriched pathways within the dominant theme, with the most frequently occurring genes representing core components of the tumor-associated transcriptional program.

#### Prediction confidence metrics

Prediction confidence was derived from class probability distributions produced by each trained model, using probabilities from the final prediction level for hierarchical classifiers. Since SVMs do not natively output probabilities, calibrated probability estimates were obtained using the internal calibration procedures implemented in scikit-learn.^26^

Prediction confidence was quantified using three complementary metrics. First, entropy of the predicted probability distribution was computed using scipy.stats.entropy, with lower values indicating higher confidence. Second, class separation was measured as the difference between the two highest class probabilities, where larger differences indicate greater prediction certainty. Third, AD distance was estimated using a modified ApplicabilityDomainDetector from the applicability_domain Python library (k = 5, α = 0.95). This approach uses Pearson correlation to assess the similarity of each sample to the training data, with larger AD distances indicating samples outside regions of reliable prediction.^42^ AD distance ratios were computed globally across all samples using the features retained after mutual-information filtering (top 20%). For each uncertainty metric, the bottom 10% of samples were flagged as uncertain or out-of-domain. Confidence was then determined based on the total number of flags: 0 flags = High confidence, 1 flag = Medium confidence, and 2–3 flags = Low confidence.

### Quantification and statistical analysis

Classification performance was evaluated using five-fold CV. Balanced accuracy was used as the primary optimization and evaluation metric to account for class imbalance, while class-specific performance was assessed using F1-scores. Performance values are reported as means across CV folds. Error bars represent standard error, standard deviation, or 95% confidence intervals as specified in the corresponding figure legends. Tissue alignment between patient samples and CLs was quantified using the tissue match fraction among the ten nearest patient samples in PCA space. Differences between patient and CL gene expression profiles were summarized using Cohen's d effect sizes. Feature selection robustness was quantified using the Jaccard index across CV folds. Gene expression specificity analyses used log2 fold changes and Pearson correlation coefficients. Pathway enrichment analysis was performed using g:Profiler. *p* values < 0.05 were considered significant. Prediction uncertainty was assessed using entropy, top-two probability difference, and AD distance.
