# Supplementary material for: Hierarchical modeling of tumor subtypes in cell lines using large-scale genomic datasets
Source: iScience. 2026 Aug 25;29(9):117315. doi: 10.1016/j.isci.2026.117315 (PMC13543817; doi:10.1016/j.isci.2026.117315)
Supplement: Supplementary file 1 [file mmc1.pdf]

iScience, Volume *29*

Supplemental information

Hierarchical modeling of tumor subtypes in cell lines using large-scale genomic datasets

Juho Mikkonen, Teemu J. Rintala, and Vittorio Fortino

SUPPLEMENTARY INFORMATION

Supplementary Table 1. Details of tissue samples from TCGA and GTEx along with the tumor subtype labels and references justifying the subtypes. Values in brackets represent the number of samples. Underlined subtypes were excluded after initial testing due to a low number of samples and poor performance. CLs were retained if (i) their lineage matched the patient tissue lineages in TCGA/GTEx, (ii) their primary disease was an exact or closely related match to a TCGA cancer type, and (iii) their disease subtypes were covered by TCGA. GDSC CLs lacking adequate metadata were excluded. Subtypes not covered by TCGA were removed. Her2 = Human epidermal growth factor receptor 2 enriched, LumA = Luminal A, LumB = Luminal B, TRU = Terminal respiratory unit, prox.-inflam = proximal inflammatory, prox.-prolif. = proximal proliferative, CIN = Chromosomal instability, MSI = Microsatellite instability, GS = Genomic stability, HM-SNV = Hypermutated-single nucleotide variants, EBV = Epstein–Barr virus–positive, G-CIMP-high = Glioma CpG island methylator phenotype high, Codel = 1p/19q-codeleted oligodendroglioma, PA-like = Pilocytic astrocytoma–like, ESCC = Esophageal squamous cell carcinoma, KIRC = Kidney renal clear cell carcinoma.

Included lineages: Lung; Skin; CNS/Brain; Esophagus/Stomach; Breast; Bowel; Head and Neck; Ovary/Fallopian Tube; Kidney; Bladder/Urinary Tract; Prostate; Adrenal Gland.

Included primary diseases: Non-Small Cell Lung Cancer; Melanoma; Diffuse Glioma; Colorectal Adenocarcinoma; Head and Neck Squamous Cell Carcinoma; Ovarian Epithelial Tumor; Invasive Breast Carcinoma; Esophagogastric Adenocarcinoma; Renal Cell Carcinoma; Bladder Urothelial Carcinoma; Esophageal Squamous Cell Carcinoma; Non-Cancerous; Prostate Adenocarcinoma; Small Bowel Cancer.

Cell line subtypes excluded (not covered by TCGA set or non-matching): Astrocytoma; Large Cell Lung Carcinoma; Lung Adenosquamous Carcinoma; Clear Cell Ovarian Cancer; Mucinous Ovarian Cancer; Endometrioid Ovarian Cancer; Anaplastic Astrocytoma; Gliosarcoma; Giant Cell Carcinoma of the Lung; Small Cell Carcinoma of the Ovary; Oligodendroglioma; Small Cell Carcinoma of the Stomach; Hyperplasia, Prostate; Immortalized MPLC Cells; Brenner Tumor, Malignant; Mixed Ovarian Carcinoma.

OncoTree Lineage	TCGA datasets / tumor types	Tumor Subtypes	Subtyping	GTEx normal primary tissues	Included CL Primary Disease(s)	TCGAbiolinks subtype column
Adrenal Gland	PCPG	Kinase signaling (68), Pseudohypoxia (61), Wnt-altered (22), Cortical admixture (22), Normal (3)	mRNA expression–based PCPG classifier (multi-omic consensus clusters)	Adrenal gland (258)	-	Subtype_mRNA
Bladder/ Urinary Tract	BLCA	squamous (144), papillary (141), infiltrated (80), Luminal (27), Neuronal (20), Normal (19)	mRNA clustering–based BLCA consensus subtypes	Bladder (21)	Bladder Urothelial Carcinoma	mRNA cluster
Bowel	READ, COAD	CIN (328), MSI (65), GS (58), Normal (51)	Genome-scale analysis: exome, DNA copy number, promoter methylation, mRNA, and microRNA	Colon sigmoid (373), Colon transverse (406)	Colorectal Adenocarcinoma, Small Bowel Cancer	Subtype_other
Breast	BRCA	LumA (565), LumB (217), Basal (190), Normal (113), Her2 (82)	PAM50	Breast mammary tissue (779)	Invasive Breast Carcinoma	Subtype_mRNA
CNS/Brain	LGG, GBM	G-CIMP-high (234), Codel (173), Mesenchymal-like (98), Classic-like (69), PA-like (26), Normal (5)	TCGA integrated methylation + mRNA–based LGG/GBM subtypes	Brain hippocampus (197), brain hypothalamus (202), brain anterior cingulate cortex_ba24 (176), brain cortex (255), brain substantia nigra (139), brain caudate basal ganglia (246), brain cerebellar hemisphere (215), brain nucleus accumbens basal ganglia (246), brain cerebellum (241), brain putamen basal ganglia (205), brain_frontal_cortex_ba9 (209), brain amygdala (152)	Diffuse Glioma	Subtype_other
Esophagus/ Stomach	ESCA, STAD	CIN (296), ESCC (90), MSI (74), GS (50), Normal (49), EBV (30)	Multi-omic STAD/ESCA consensus classification	Esophagus muscularis (515), stomach (359), esophagus gastroesophageal junction (375), esophagus mucosa (555)	Esophagogastric Adenocarcinoma	Subtype_other
Head and Neck	HNSC	Basal (87), Mesenchymal (75), Atypical (68), Classical (49), Normal (44)	mRNA expression based	Minor salivary gland (162)	Head and Neck Squamous Cell Carcinoma	Subtype_mRNA
Kidney	KICH, KIRP, KIRC	KIRC (534), Normal (129), Type 1 Papillary RCC (75), Type 2 Papillary RCC (60), Eosin.0 (47)	mRNA + CNV based	Kidney medulla (4), kidney cortex (85)	Renal Cell Carcinoma	KICH: Subtype_other, KIRP: tumor_type.KIRP.path.
Lung	LUSC, LUAD	Normal (110), TRU (97), prox.-inflam (88), classical (65), prox.-prolif. (63), secretory (44), basal (43), primitive (27)	mRNA expression-based subtypes derived from TCGAbiolinks	Lung (578)	Non-Small Cell Lung Cancer	LUSC: Subtype_mRNA, LUAD: expression_subtype
Ovary/ Fallopian Tube	OV	Proliferative (116), Differentiated (108), Mesenchymal (98), Immunoreactive (90)	Subtype mRNA	Ovary (459)	Ovarian Epithelial Tumor	Subtype_mRNA
Prostate	PRAD	1-ERG (152), Normal (52), 5-SPOP (39), 2-ETV1 (28)	Gene-fusion and molecular subtype	Prostate (245)	Prostate Adenocarcinoma	Subtype_mRNA
Skin	SKCM	immune (168), keratin (102), MITF-low (59), Normal (1)	mRNA based	Skin sun exposed lower leg (701), skin not sun exposed suprapubic (604)	Melanoma	Subtype_mRNA

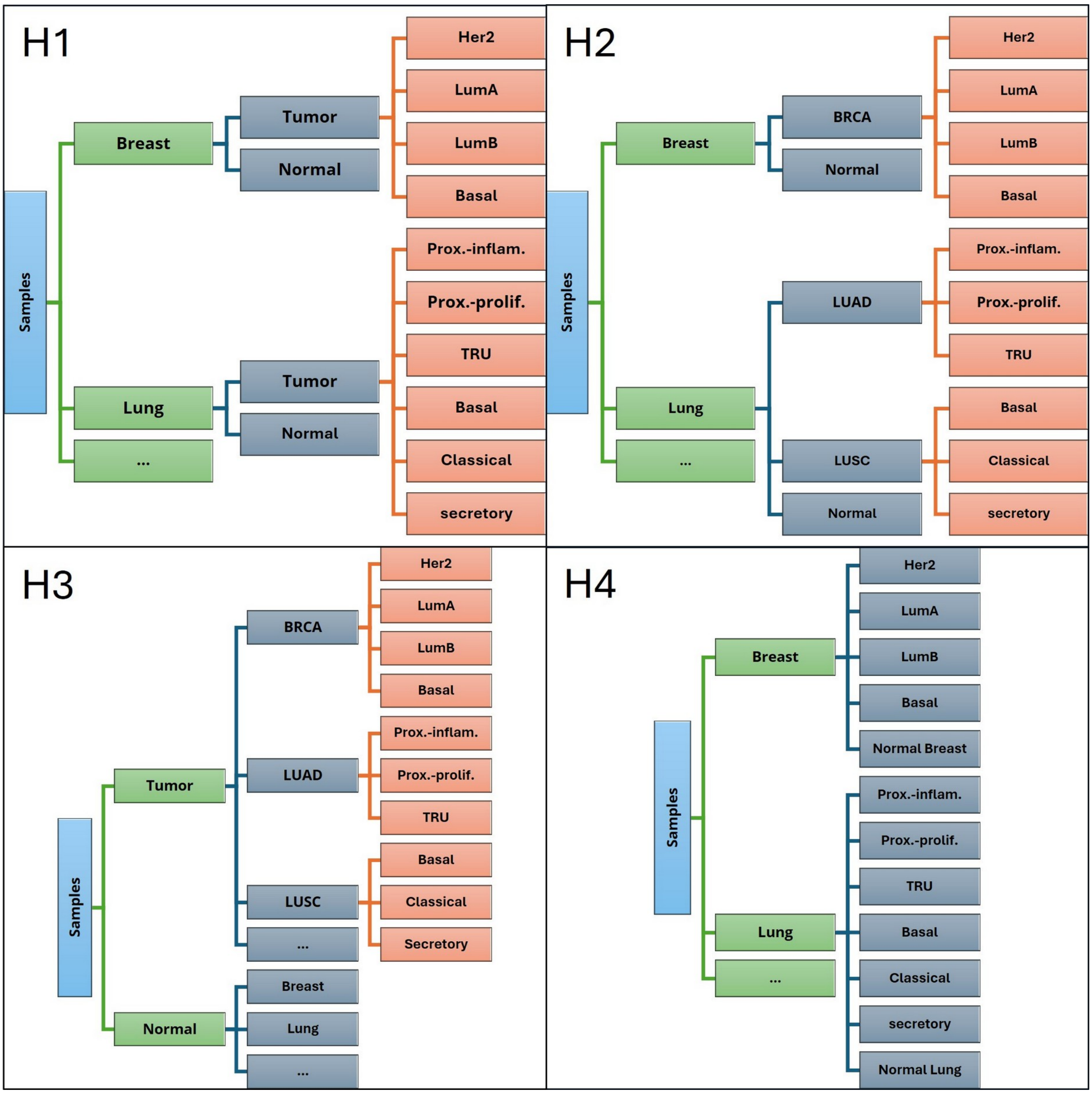

Supplementary Figure 1. Example visualizations of the different hierarchies used for training hierarchical models. (Hierarchies contain only breast and lung categories for visualization purposes).

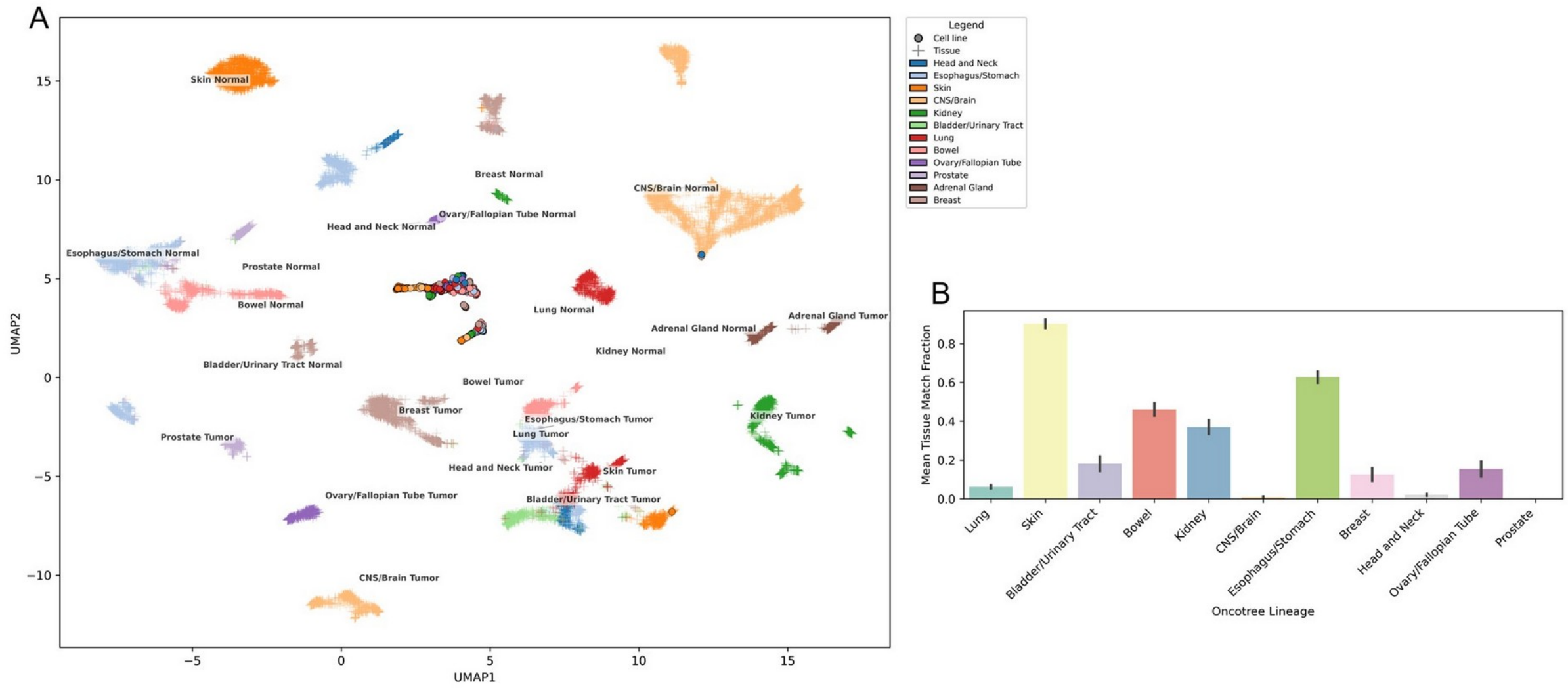

Supplementary Figure 2. Uncorrected data UMAP and tissue match. Mean tissue match (10 nearest neighbours) and UMAP of the uncorrected RNA-seq data.

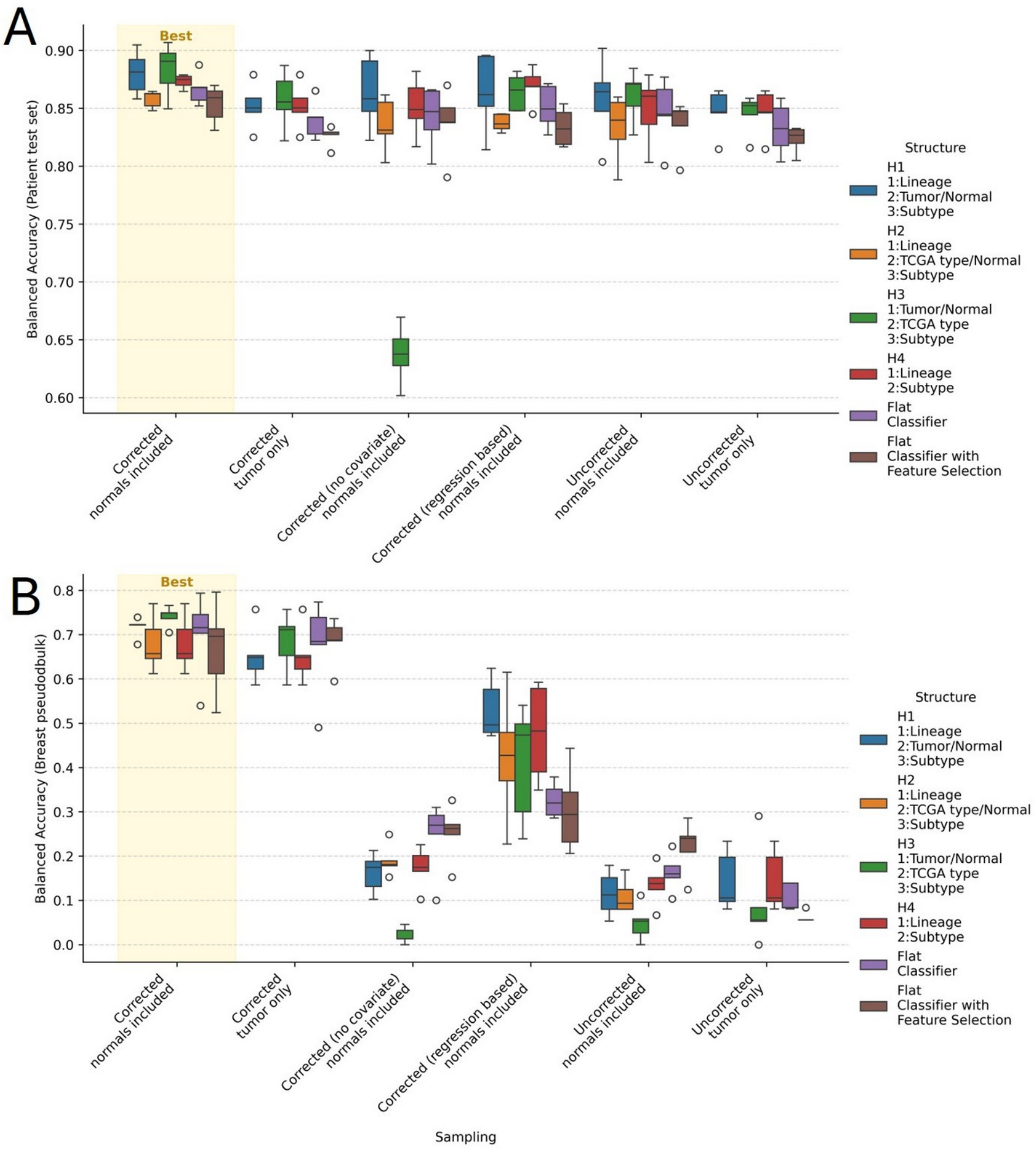

Supplementary Figure 3. Ablation benchmarking results of HC systems for cancer subtype matching. (A) Balanced accuracy of the Logistic Regression (L2-regularized) inner classifier across different hierarchical classification architectures and their corresponding flat classifier counterparts, evaluated with and without mRMR-based feature selection on the patient test dataset (5-fold cross-validation). Performance was compared using ComBat batch-corrected and uncorrected data, with normal tissue samples either included or excluded from model training. Inclusion of normal samples consistently improved classification performance, particularly when combined with batch correction. Importantly, batch correction did not artificially increase performance

when predictions were restricted to tumor samples from TCGA. **(B)** Balanced accuracy of the same models evaluated on an external breast cancer pseudobulk dataset (5-fold cross-validation). Batch correction was essential for achieving robust performance on external data, resulting in substantial improvements in balanced accuracy. Inclusion of normal samples provided modest additional gains compared with the effect of batch correction alone.

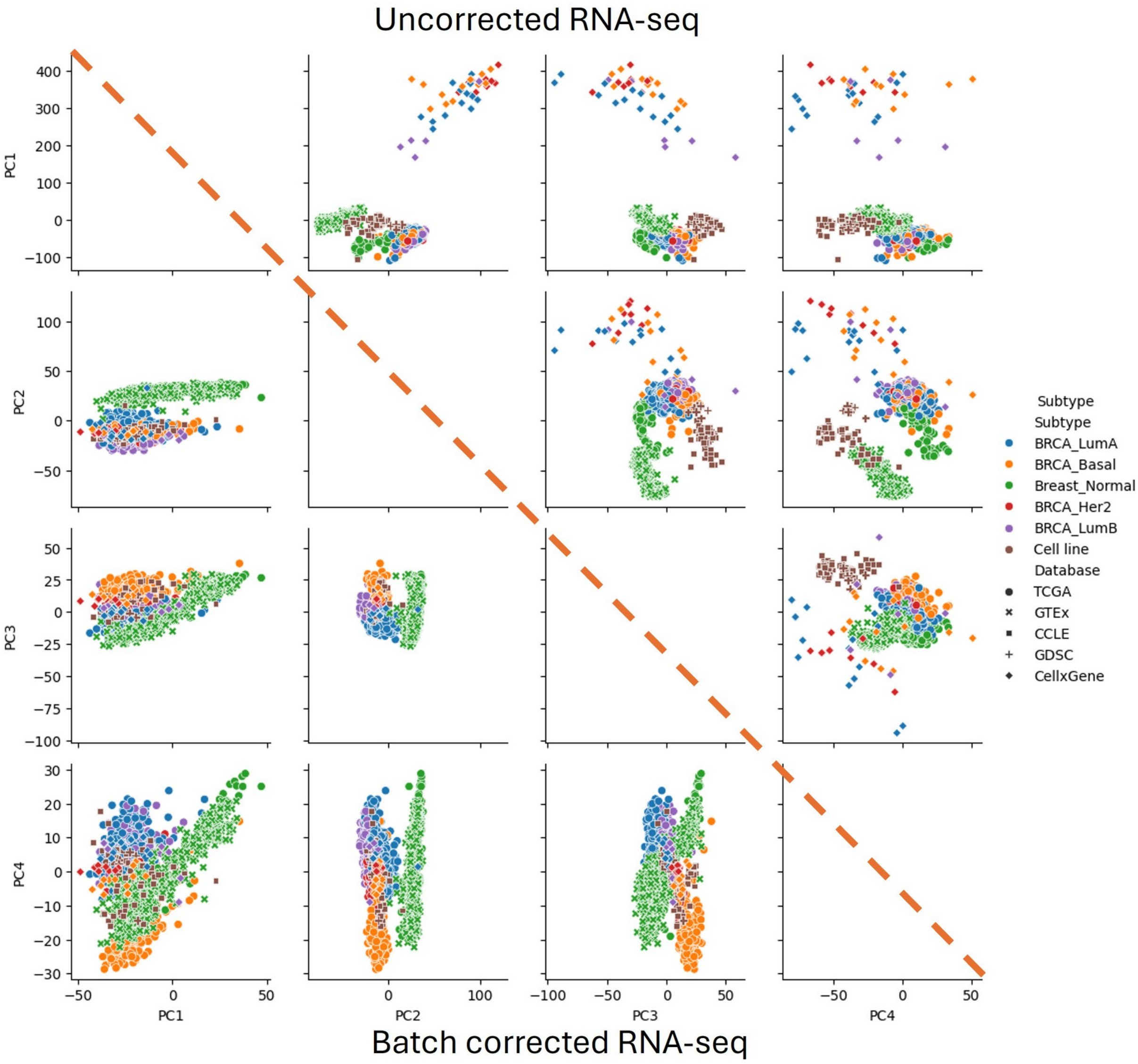

Supplementary Figure 4. PCA plot showing the effect of the batch correction (ComBat) on breast cancer samples from TCGA, healthy individuals from GTEx, pseudobulk samples derived from scRNA from CellxGene and CLs from CCLE and GDSC. Samples show grouping by database before correction, and by subtype after correction.

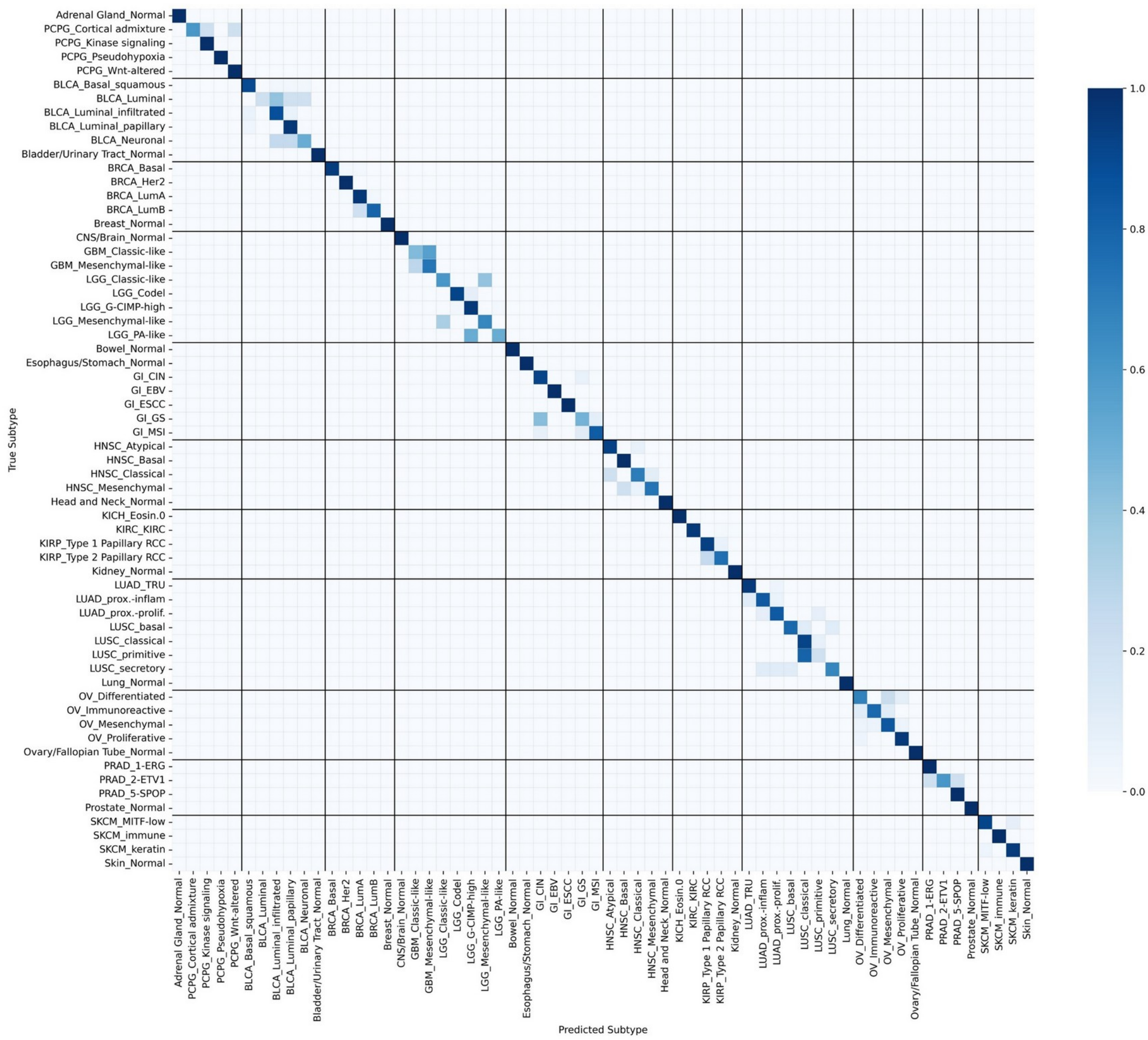

Supplementary Figure 5. Confusion matrix of the best model (logistic regression with L2 penalty using H3: Tumor vs. Normal → TCGA Cancer Type → Tumor Subtype. Results from cross-validated patient test set (CV=5). Normalized. Labels contain TCGA type or lineage (for non-cancerous samples) and subtype separated using an underscore.

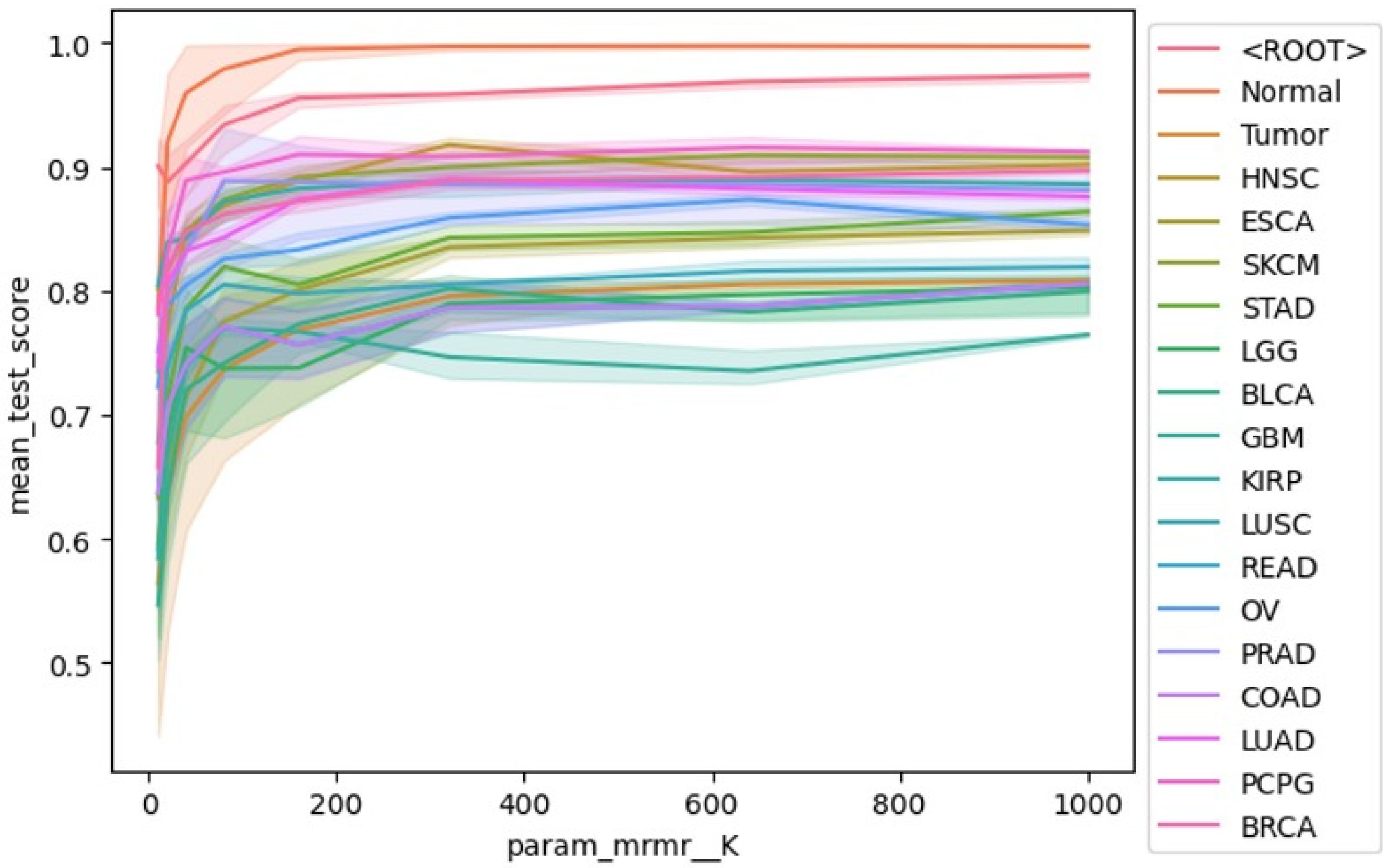

Supplementary Figure 6. Mean test score of individual nodes during nested model tuning for optimal number of features (cv=5). Tuning was done on batch corrected TCGA and GTEx data. Tested feature set sizes were: 10, 20, 40, 80, 160, 320, 640, 1000. Logistic regression (L2) model with hierarchy H3.

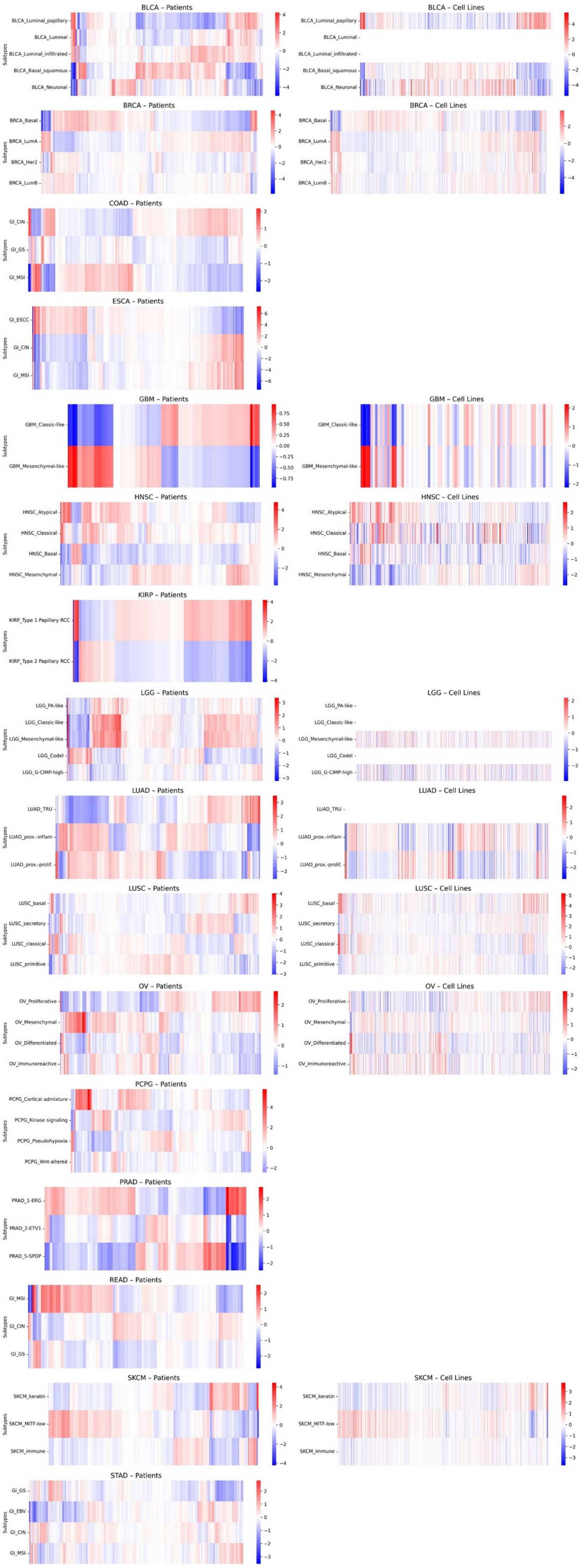
BLCA – Patients
BLCA_Luminal_papillary
BLCA_Luminal
BLCA_Luminal_infiltrated
BLCA_Basal_squamous
BLCA_Neuronal
BLCA – Cell Lines
BRCA – Patients
BRCA_Basal
BRCA_LumA
BRCA_Her2
BRCA_LumB
BRCA – Cell Lines
COAD – Patients
GI_CIN
GI_GS
GI_MSI
ESCA – Patients
GI_ESCC
GBM – Patients
GBM_Classic-like
GBM_Mesenchymal-like
GBM – Cell Lines
HNSC – Patients
HNSC_Atypical
HNSC_Classical
HNSC_Basal
HNSC_Mesenchymal
HNSC – Cell Lines
KIRP – Patients
KIRP_Type 1 Papillary RCC
KIRP_Type 2 Papillary RCC
LGG – Patients
LGG_PA-like
LGG_Classic-like
LGG_Mesenchymal-like
LGG_Codel
LGG_G-CIMP-high
LGG – Cell Lines
LUAD – Patients
LUAD_TRU
LUAD_prox.-inflam
LUAD_prox.-prolif.
LUAD – Cell Lines
LUSC – Patients
LUSC_basal
LUSC_secretory
LUSC_classical
LUSC_primitive
LUSC – Cell Lines
OV – Patients
OV_Proliferative
OV_Mesenchymal
OV_Differentiated
OV_Immunoreactive
OV – Cell Lines
PCPG – Patients
PCPG_Cortical admixture
PCPG_Kinase signaling
PCPG_Pseudohypoxia
PCPG_Wnt-altered
PRAD – Patients
PRAD_1-ERG
PRAD_2-ETV1
PRAD_5-SPOP
READ – Patients
SKCM – Patients
SKCM_keratin
SKCM_MITF-low
SKCM_immune
SKCM – Cell Lines
STAD – Patients
GI_EBV
Subtypes

Supplementary figure 7. Log2 fold change (log2FC) comparison of subtype-specific gene expression in patient tumors (left panels) **and corresponding CLs** (right panels). Rows represent tumor subtypes. Columns represent genes selected by the best model for each TCGA type, ordered according to hierarchical clustering on patient data. Only cancer types with CL samples representing at least two subtypes are shown in paired panels. CL subtypes determined based on predictions by the best performing ML model. Color intensity indicates the direction and magnitude of subtype-associated expression differences.

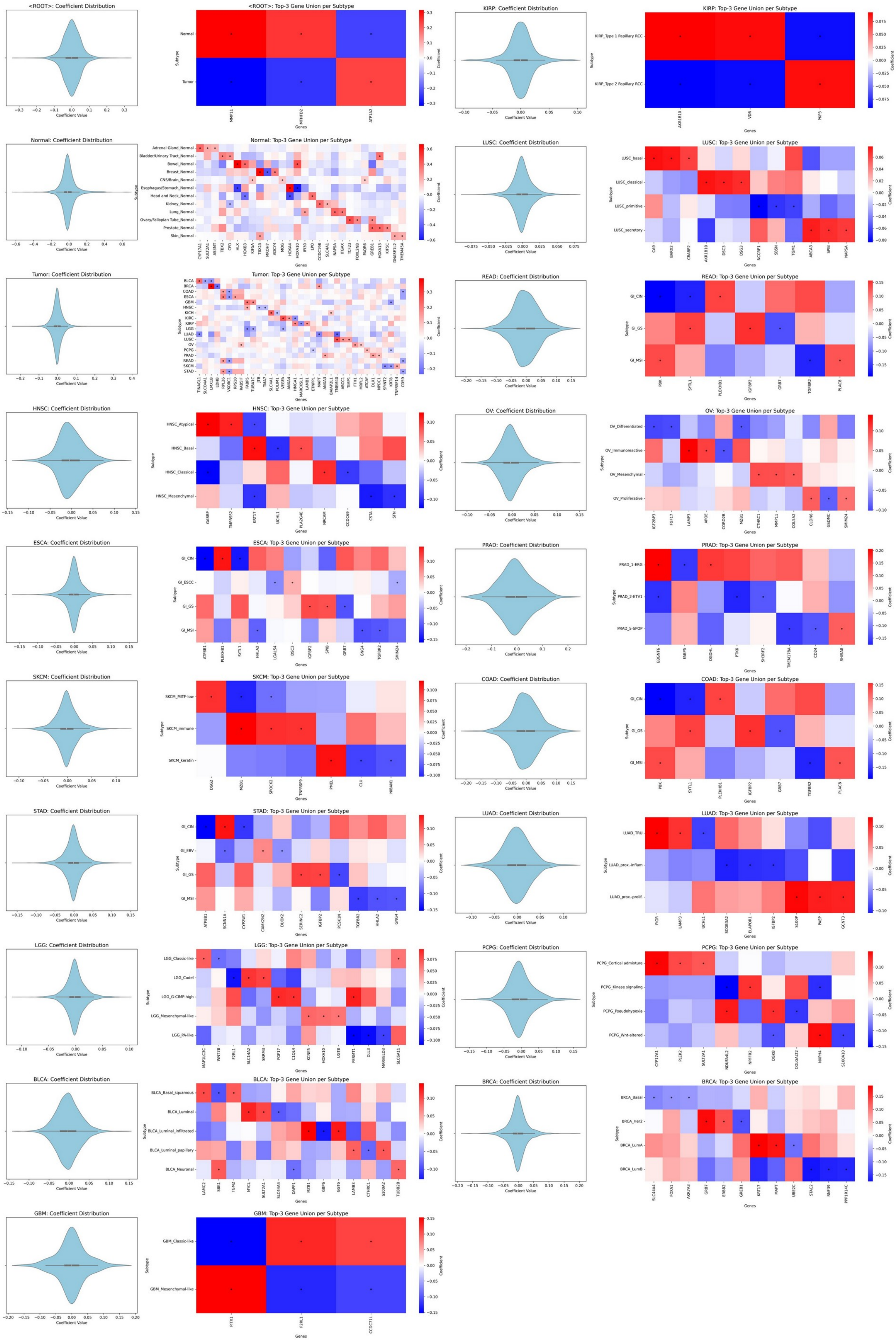

<ROOT>: Coefficient Distribution
<ROOT>: Top-3 Gene Union per Subtype
KIRP: Coefficient Distribution
KIRP: Top-3 Gene Union per Subtype
Normal: Coefficient Distribution
Normal: Top-3 Gene Union per Subtype
LUSC: Coefficient Distribution
LUSC: Top-3 Gene Union per Subtype
Tumor: Coefficient Distribution
Tumor: Top-3 Gene Union per Subtype
READ: Coefficient Distribution
READ: Top-3 Gene Union per Subtype
HNSC: Coefficient Distribution
HNSC: Top-3 Gene Union per Subtype
OV: Coefficient Distribution
OV: Top-3 Gene Union per Subtype
ESCA: Coefficient Distribution
ESCA: Top-3 Gene Union per Subtype
PRAD: Coefficient Distribution
PRAD: Top-3 Gene Union per Subtype
SKCM: Coefficient Distribution
SKCM: Top-3 Gene Union per Subtype
COAD: Coefficient Distribution
COAD: Top-3 Gene Union per Subtype
STAD: Coefficient Distribution
STAD: Top-3 Gene Union per Subtype
LUAD: Coefficient Distribution
LUAD: Top-3 Gene Union per Subtype
LGG: Coefficient Distribution
LGG: Top-3 Gene Union per Subtype
PCPG: Coefficient Distribution
PCPG: Top-3 Gene Union per Subtype
BLCA: Coefficient Distribution
BLCA: Top-3 Gene Union per Subtype
BRCA: Coefficient Distribution
BRCA: Top-3 Gene Union per Subtype
GBM: Coefficient Distribution
GBM: Top-3 Gene Union per Subtype

Supplementary Figure 8. Summary of classifier coefficients for all nodes in the hierarchical model. For each node, the left panel shows the distribution of all coefficients from the associated classifier, illustrating the overall strength and variability of model weights. The right panel displays the coefficients for the union of the top three genes identified independently for each class in the node. Coefficients for all classes are shown for these genes, enabling comparison of subtype-specific and shared patterns. Dots indicate genes that were ranked among the top three most influential features for the corresponding class. Together, these visualizations highlight the key genes driving subtype discrimination and reveal how classifier importance differs across subtypes and cancer types.

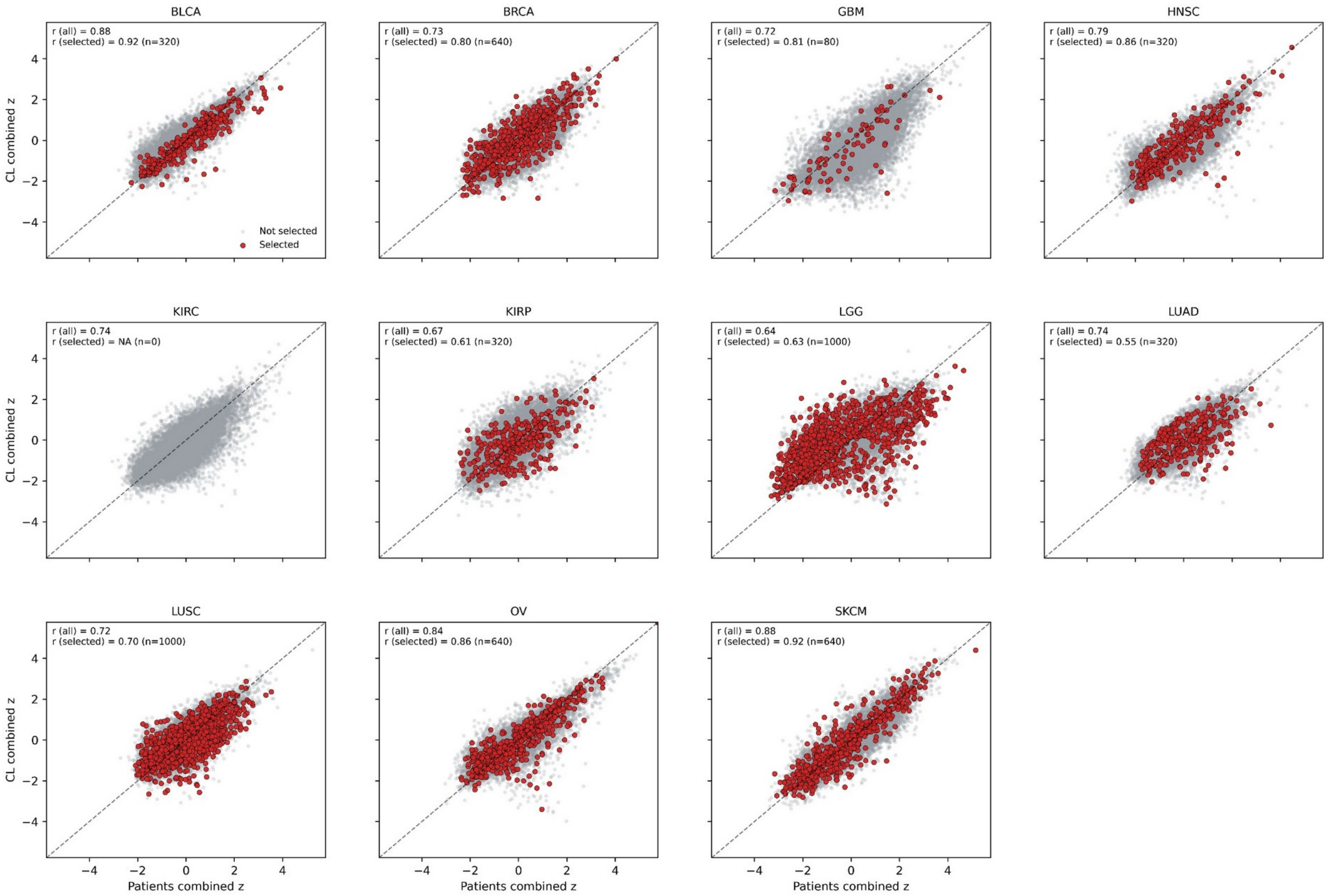

Supplementary Figure 9. Concordance of combined z-scores between patient tumors and CLs by TCGA cancer type. Grey = all genes, red = model-selected genes. For each panel, Pearson correlations (r) are shown for all genes and for model selected genes.

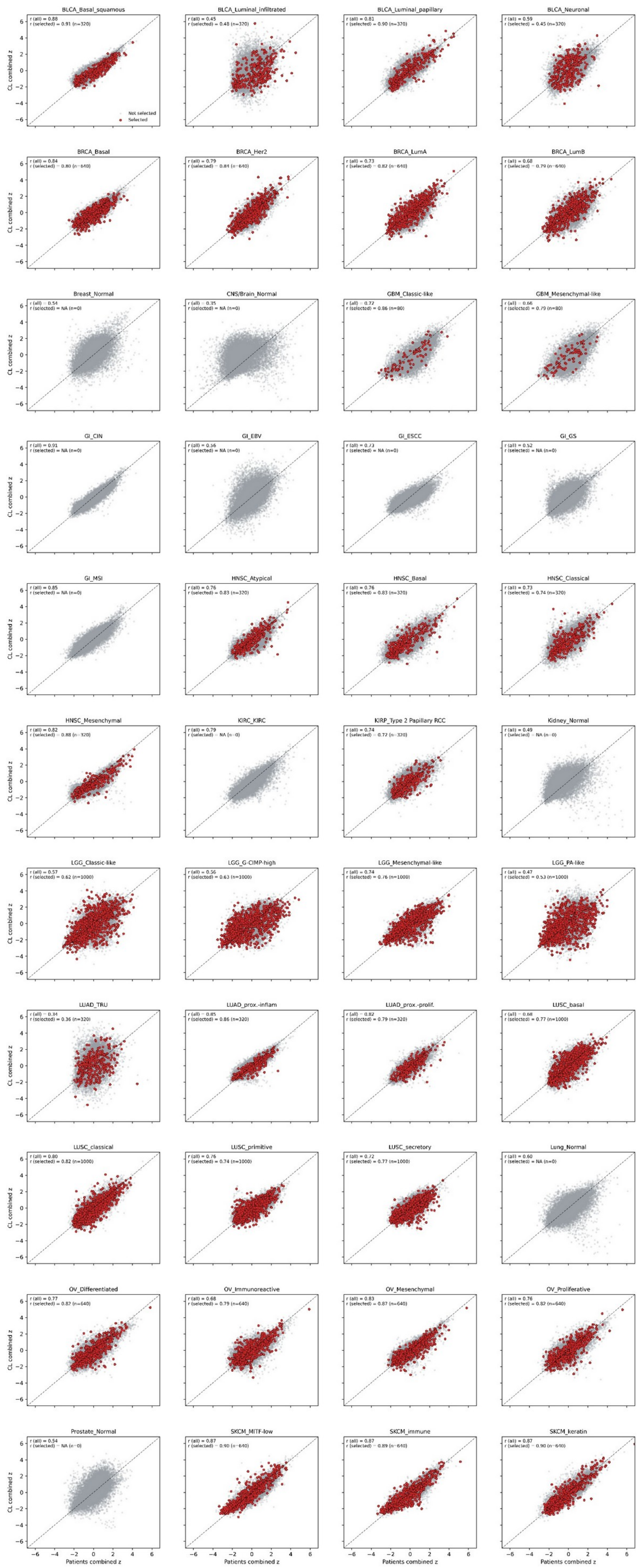
BLCA_Basal_squamous
BLCA_Luminal_infiltrated
BLCA_Luminal_papillary
BLCA_Neuronal
BRCA_Basal
BRCA_Her2
BRCA_LumA
BRCA_LumB
Breast_Normal
CNS/Brain_Normal
GBM_Classic-like
GBM_Mesenchymal-like
GI_CIN
GI_EBV
GI_ESCC
GI_GS
GI_MSI
HNSC_Atypical
HNSC_Basal
HNSC_Classical
HNSC_Mesenchymal
KIRC_KIRC
KIRP_Type 2 Papillary RCC
Kidney_Normal
LGG_Classic-like
LGG_G-CIMP-high
LGG_Mesenchymal-like
LGG_PA-like
LUAD_TRU
LUAD_prox.-inflam
LUAD_prox.-prolif.
LUSC_basal
LUSC_classical
LUSC_primitive
LUSC_secretory
Lung_Normal
OV_Differentiated
OV_Immunoreactive
OV_Mesenchymal
OV_Proliferative
Prostate_Normal
SKCM_MITF-low
SKCM_immune
SKCM_keratin
Not selected
Selected
CL combined z
Patients combined z

Supplementary Figure 10. Concordance of combined z-scores between patient tumors and CLs by subtype. Grey = all genes, red = model-selected genes. For each panel, Pearson correlations (r) are shown for all genes and for model selected genes. Subtype gene sets were mapped to TCGA parent types.

Supplementary Table 2. Analysis of top 3 selected genes for each node. Provides a literature-based rationale for their validity as biomarkers for various cancers.

Gene	Top Nodes	Rationale	References (DOI)
ADIRF	BLCA, BRCA, GBM, LUSC	Linked to poor prognosis in breast cancer and is implicated to play a role in multiple other cancer types. Part of 12 gene prognostic risk score panel for triple negative breast cancer.<br>Downregulation associated with malignant transformation of lung cancer cells, implying tumor suppressor role.	https://doi.org/10.21037/tcr-23-1554<br><br>https://doi.org/10.1007/s00204-023-03608-y
ASRGL1	LUAD	Has been found to correlate negatively with overall survival in lung cancer patients.	https://doi.org/10.3389/fonc.2021.680070
BIN1	PCPG	Tumor suppressor regulating cancer progression and tumor microenvironment. Recently connected to pheochromocytoma. Not formally associated with paraganglioma.	https://doi.org/10.1016/j.compbiomed.2025.110862
CCDC69	HNSC	Associated with survival prognosis in HNSC. Expression often reduced in various cancers and related to poor outcomes.	https://doi.org/10.1007/s12672-025-03127-5<br><br>https://doi.org/10.3389/fsurg.2022.879921
CD300LG	Tumor or Normal	Reduced expression of CD300LG is associated with tumor progression, metastasis, and poorer survival outcomes.	https://doi.org/10.1002/cam4.4905<br><br>https://doi.org/10.1007/s10238-025-01690-3
CRISPLD2	OV	Functions as an anti-inflammatory protein. Associated with prognosis in OV.	https://doi.org/10.1186/s12935-020-01361-1
CSTA	HNSC	CSTA linked with better HNSC prognosis, via inhibiting metastasis of nasopharyngeal carcinoma cells through suppressing AKT signalling.	https://doi.org/10.1016/j.bbadis.2023.166696
CYP17A1	PCPG	Regulator of steroidogenesis. Higher expression linked to normal adrenocortical cells.	https://doi.org/10.7554/eLife.87586
DDX27	COAD, READ, STAD	DDX27 is known to promote growth, metastasis, and chemoresistance particularly in GI cancers. Roles in RNA metabolism and various cellular processes such as RNA splicing, translation and ribosome biogenesis. Strong links to tumorigenesis. Also included in the Tumor or Normal, and All Tumor nodes.	https://doi.org/10.1186/s12967-025-07004-1
DRP2	STAD	Role in peripheral nervous system. Linked to poor prognosis in breast, lung and brain cancer. Potential novel marker for tumors in the stomach.	https://doi.org/10.1186/s11658-019-0162-0
EEF1G	All Normal	Role in the enzymatic delivery of aminoacyl tRNAs to ribosomes. Widely expressed over various tissues, especially the brain and GI.	Human protein atlas: http://dx.doi.org/10.1126%2Fscience.1260419
EPO	Tumor or Normal	Often overexpressed in various cancers, promoting tumor growth, survival, angiogenesis, and resistance.	https://doi.org/10.3389/fimmu.2014.00563
ESCO2	STAD	Roles in cell cycle and proliferation regulation. Linked to poor prognosis pan-cancer	https://doi.org/10.1186/s12885-024-12213-w<br>https://doi.org/10.3389/fmolb.2022.1022056
EVA1B	BLCA, LUSC, PCPG	EVA1B has been indicated as an oncogene in various cancers. Expression is correlated with immune cell infiltration among other immune system activating effects. Associated with poor survival outcomes in various cancers such as colorectal cancer and glioma.	https://doi.org/10.3389/fimmu.2022.809837<br><br>https://doi.org/10.3389/fimmu.2021.648416
FAM72C	OV	Paralog of gene family that is specific to neural stem cells. Linked to predictive capability in precancerous lesions of inflammatory bowel disease and colorectal cancer. No mention of association with ovarian cancer in paralog C. FAM72B associated with prognosis in ovarian cancer. Potential novel marker.	https://doi.org/10.1016/j.bbrep.2023.101506
FOXA1	BRCA, HNSC	A well-established luminal marker in breast cancer. Selected for BRCA, HNSC, BLCA, LUSC, LUAD, and STAD. Its role in subtype differentiation and prognosis has been documented across all these cancers.	https://doi.org/10.1016/j.gene.2017.11.023<br>https://doi.org/10.1371/

			JOURNAL.PONE.0036669<br>https://doi.org/10.1038/S41419-022-05150-8;TECHMETA<br><br>https://doi.org/10.3390/CANCERS14194699
GMFG	LUAD	Roles in immune function, neural development and angiogenesis. Known oncogene and associated with poor prognosis and metastasis in various cancers. Shown to be fundamental in p53 signalling and inhibiting cancer progression in LUAD. High expression related to malignancy in triple negative breast cancer.	https://doi.org/10.1080/21655979.2022.2049958<br><br>https://doi.org/10.3390/genes14061157
HHIP	COAD, READ	Known tumor suppressor in various cancers, especially gastric cancers. Low levels associated with metastasis, immune infiltration and poor prognosis.	https://doi.org/10.1186/s12876-023-02867-4
KIF4B	Tumor or Normal	KIF4B is part of the kinesin superfamily known for intracellular transport. Reported to be a high-risk gene in the majority of TCGA tumors. Low expression across tumor types.	https://doi.org/10.3389/fonc.2023.1179897
KRT7	KIRP	Expressed during differentiation of epithelial tissues. Overexpression promotes tumor progression. Used as diagnostic marker in tumor pathology due to presence in particular tumors. Low expression overall in renal cancer but has been shown to be able to distinguish between different types of renal cancer.	https://doi.org/10.3389/fonc.2023.1243871<br><br>https://doi.org/10.1007/s00428-012-1209-z
LGALS4	ESCA	Specific to gastrointestinal tract in healthy individuals. Detected in various cancers within and outside the GI, like breast and lung. Linked to tumor stage in CRC.	https://doi.org/10.1007/s13238-016-0262-9
LMX1B	All Tumor	High tissue specificity. Expressed in the proximal digestive tract, brain and kidney. Highly expressed in breast cancer.	Human protein atlas: http://dx.doi.org/10.1126%2Fscience.1260419
MBP	ESCA	Suppressor of the c-Myc protein. Seen to have a suppressive effect on tumor progression in gastric cancer CLs. Competitive binding to c-Myc via lncRNA has shown potential as an antitumor therapy in ESCC.	https://doi.org/10.1091/mbc.e09-05-0386<br><br>https://doi.org/10.1158/1541-7786.MCR-20-1025
MMP11	ESCA	Encodes enzyme to break down extracellular matrix. Has stimulating effect on tumor growth and has been observed in most cancers. Seen to play a key role in ESCA progression though contributing to immune evasion. Potential immunotherapy target in ESCA.	https://doi.org/10.1016/j.tranon.2025.102604
NUF2	LUAD	Component of nuclear division. Well-characterized immunological and prognostic marker of LUAD, correlated with advanced stage and poor survival. Silencing as been seen to halt tumor progression. Shows promise as therapeutic target.	https://doi.org/10.21037/jtd-2025-704<br><br>https://doi.org/10.3389/fonc.2022.795971<br><br>https://doi.org/10.1155/2022/1161931
OTX1	PRAD	Homeabox protein associated with prostate cancer risk. Main function is brain development.<br>OTX1 expression levels associated with PSA levels in men without prostate cancer. PSA is routinely used as a marker of PRAD and PRAD progression.	https://doi.org/10.1371/journal.pgen.1004102<br><br>https://doi.org/10.1101/2023.05.04.23289526
PLG	All Normal	Expression is specific to liver, kidney and bladder tissues. Thus, a strong marker for separating non-cancerous tissues from one another.	Human protein atlas: http://dx.doi.org/10.1126%2Fscience.1260419
PXN	PRAD	Encodes paxisillin, a protein mediating cell adhesion, migration and AR and ERK signalling. Over-expression has been shown to increase cell migration in prostate cancer, leading to metastasis. Knock-down inhibits tumor growth.	https://doi.org/10.1172/JCI62044
RAB34	LGG	RAB34 has been identified as a potential biomarker for distinguishing cancer subtypes or tumor severity in LGG and has been shown to be correlated with inflammatory activities, immune infiltration, and immune checkpoints in glioma. Additionally, inhibition of RAB34 has been shown to restrain the	https://doi.org/10.1042/BSR20212624

		growth and invasion of glioma cells and reverse the EMT process.	
RASGRP2	KIRP	Is critical in platelet activation and aggregation. Has been reported as a potential biomarker for LUAD and STAD due to correlation with pathologic stage, overall survival and progression free interval.	https://doi.org/10.3389/fimmu.2023.1100231
REEP2	LGG	REEP2 is highly expressed in the brain and in gliomas. It is closely associated with various neurodegenerative diseases, and it has been reported to function as an unfavorable prognostic marker in glioma. This is reversed for melanoma and breast cancer.<br><br>REEP4, another gene in the same family has been proposed as a key factor in the malignant process of LGG.	Human protein atlas: http://dx.doi.org/10.1126%2Fscience.1260419<br><br>https://doi.org/10.18632/aging.205695
RPL36A	All Tumor	An oncogene frequently upregulated in CRC, breast and lung cancers. Proposed to activate the ERK pathway to promote cancer growth in CRC. Knockdown has tumor suppressive effect, proposing a prospective therapeutic target.	https://doi.org/10.1016/j.tranon.2024.102170
RPS10	All Tumor	Involved in ribosome biogenesis and function. Thus, it is expressed in most tissues. Upregulation has been reported in various cancers, and	https://doi.org/10.3389/fonc.2022.835549
RPS17	All Normal	Similar to RPS10, RPS17 is expressed in most tissues with varying levels, and is upregulated especially in gastric cancer.	https://doi.org/10.3389/fonc.2022.835549
SAA2	PRAD	SAA family of proteins have been associated with various cancers and show prognostic value. In PRAD, it is often upregulated in severe cases that metastasize to the bone.	Human protein atlas: http://dx.doi.org/10.1126%2Fscience.1260419<br><br>https://doi.org/10.1373/clinchem.2004.041087
SPOCK2	SKCM	SPOCK2 regulates cell differentiation and growth. It has previously been reported to play an important role in cancer. Recently, it has been reported to regulate tumor progression in melanoma with knockdown inducing notable malignant progression and tumor growth.	https://doi.org/10.1111/exd.15092
SPP2	KIRP	Encodes an extracellular matrix protein primarily in the liver and bone with some expression in the kidney. Particularly high expression in kidney cell lines, implying some role in kidney cancer. SPP2 has been seen to be lowly expressed in tumors, perhaps suggesting a role as a tumor suppressor.<br><br>Expression of SPP2 has been associated with future decline in renal function.	Human protein atlas: http://dx.doi.org/10.1126%2Fscience.1260419<br><br>https://doi.org/10.1186/s12885-019-6441-3<br><br>https://doi.org/10.1038/s41598-025-28988-2
TNS4	SKCM	Predicted to enable actin binding activity. Enhances cell survival in cancer by promoting the epithelial-mesenchymal transition. Strong expression has been linked to worse survival rates for melanoma patients and has been proposed as a prognostic marker.	https://doi.org/10.1371/journal.pone.0080492
TSPAN1	COAD, READ	Regulates cell development, activation, growth and motility. Well known biomarker for colorectal cancer. Correlated with histological grade, metastasis and tumor stage. Overexpression associated with significantly shortened lifespan in colorectal cancer patients.	https://doi.org/10.3748/wjg.15.2270
UNC93B1	OV	Involved in innate and adaptive immune responses. Associated with various cancers with female cancers having the strongest association in TCGA data.	https://doi.org/10.1093/nar/gkae1050
UPK2	BLCA	UPK2 is a relatively specific marker of bladder cancer. It is also expressed in some CRCs and has been shown to be able to identify aggressive cases. UPK2 has also been reported to distinguish between molecular subtypes with luminal and basal characteristics in bladder cancer.	https://doi.org/10.1038/s41416-025-03300-1<br><br>https://doi.org/10.1073/pnas.1318376111
UTP14C	GBM, LGG, LUSC, SKCM	Involved in meiotic cell cycle. Important for spermatogenesis and rRNA processing. Highly expressed in GBM. Little research on the relationship of UTP14C with cancer exists, though it has been suggested to share a downregulating effect on TP53, due to structural similarity to UTP14A (associated with cancer progression in melanoma and CRC). Also, UTP14C has been linked with resistance to therapy in cancer, implying a poor prognosis. Potential target for further research.	https://doi.org/10.21203/rs.3.rs-3258063/v1<br><br>https://doi.org/10.1002/advs.202207357<br><br>https://doi.org/10.1016/j.canlet.2018.10.010
ZNF593	BRCA, GBM	ZNF593 is highly expressed in BRCA and is correlated with a poor prognosis due to an association with cancer progression and development of drug resistance. Promotes tumor growth via influencing DNA repair and cell-cycle	https://doi.org/10.1016/j.isci.2024.111513

		regulation.<br><br>ZNF593 lacks research in GBM but it has been suggested as significant in predicting survival in GBM patients.	https://doi.org/10.1158/0008-5472.CAN-06-1408
